# Genetic Homogeneity Revealed Using SCoT, ISSR and RAPD Markers in Micropropagated *Pittosporum eriocarpum* Royle- An Endemic and Endangered Medicinal Plant

**DOI:** 10.1371/journal.pone.0159050

**Published:** 2016-07-19

**Authors:** Julie Thakur, Mayank D. Dwivedi, Pragya Sourabh, Prem L. Uniyal, Arun K. Pandey

**Affiliations:** Department of Botany, University of Delhi, Delhi, India; United States Department of Agriculture, UNITED STATES

## Abstract

*Pittosporum eriocarpum* Royle, a medicinally important taxon, is endemic to Uttarakhand region of Himalaya. It has become endangered due to over-collection and the loss of habitats. As raising plants through seeds in this plant is problematic, a reliable protocol for micropropagation using nodal explants has been developed. High shoot regeneration (95%) occurred in MS medium augmented with BA 0.4mg/l in combination IBA 0.6mg/l. *In vitro* regenerated shoots were rooted in MS medium supplemented with three auxins, of which 0.6 mg/l indole butyric acid proved to be the best for rooting (90%) with maximum number of roots per shoot. Thereafter, rooted plants were hardened and nearly 73% of rooted shoots were successfully acclimatized and established in the field. Start codon targeted (SCoT), inter simple sequence repeats (ISSR) and random amplified polymorphic DNA (RAPD) markers were used to validate the genetic homogeneity amongst nine *in vitro* raised plantlets with mother plant. DNA fingerprints of *in vitro* regenerated plantlets displayed monomorphic bands similar to mother plant, indicating homogeneity among the micropropagated plants with donor mother plant. The similarity values were calculated based on SCoT, ISSR and RAPD profiles which ranged from 0.89 to 1.00, 0.91 to 1.00 and 0.95 to 1.00 respectively. The dendrograms generated through Unweighted Pair Group Method with arithmetic mean (UPGMA) analysis revealed 97% similarity amongst micropropagated plants with donor mother plant, thus confirming genetic homogeneity of micropropagated clones. This is the first report on micropropagation and genetic homogeneity assessment of *P*. *eriocarpum*. The protocol would be useful for the conservation and large scale production of *P*. *eriocarpum* to meet the demand for medicinal formulations and also for the re-introduction of *in vitro* grown plants in the suitable natural habitats to restore the populations.

## Introduction

*Pittosporum eriocarpum* (Pittosporaceae), commonly known as Agni [1]**,** is an endangered [2] and endemic medicinal tree species of Uttarakhand region occurring at an altitudinal range of 1000-2200m [3]. It is represented by a few populations in lesser Himalayan range, including Mussoorie hills, Doon valley, Dehradun, Dhanlagiri, Chamba, Nagli in Tehri, Kurakhad, Jeullikot and Nainital [4,5]. It is reported to occur mostly on the calcium rich rocky slopes in exposed sites [6]. It is a small evergreen tree up to 5m height with alternate or whorled leaves. Flowers are pale yellow, which remain in many umbellate corymbs. Fruit is a globose capsule (10-12mm) with 8–10 blackish-red seeds. The bark is aromatic and used in bronchitis, and as expectorant and febrifuge. Application of root paste provides relief in rheumatic swelling [3]. It is also used as fodder. The tree is suitable for soil conservation and reclamation of degraded sites [4].

Micropropagation is an efficient and alternative method for large scale production of endangered plants in a short period of time. Effect of IBA on rooting in *P*. *tobira* var. *variegatum* is reported to be insignificant [7]. Plant regeneration from nodal region of *P*. *napaulensis* (DC.) Reader & Wilson has been reported [8]. *In vitro* seed germination followed by hypocotyl sectioning to raise plants has been documented in *P*. *tobira* [9]

*P*. *eriocarpum* can be regenerated asexually by layering or sowing [4], but such regenerates require periodic moist and dry conditions in moderate cool temperature. The seed coat is surrounded by thick gummy substance which delays seed germination. There is an urgent need to produce more plants through micropropagation which has advantage over conventional methods [10].

Micropropagation is assumed to cause a change in genetic makeup of plant tissue and can lead to DNA sequence variation and generation of somaclonal variation [11]. Thus, it is a major issue of concern in micropropagation, where the regenerates are expected to be homogenous to donor mother plant (MP). To overcome this, it is a prerequisite to validate the genetic homogeneity to authenticate the quality of micropropagated plants for commercial use. The genetic homogeneity amongst the micropropagated plants with mother plant has been assessed by PCR based molecular markers, i.e., AFLP (Amplified Fragment Length Polymorphism), ISSR (Inter Simple Sequence Repeats), RAPD (Random amplified polymorphic DNA), SCoT (Start codon targeted) and SSR (Simple Sequence Repeats) [12–14].

The goal of this research was to provide an efficient and reproducible method for *in vitro* propagation of *P*. *eriocarpum* via nodal explants for large scale production and assessment of genetic homogeneity of micropropagated plantlets through SCoT, ISSR and RAPD markers. This objective was achieved by healthy material collection, *in vitro* nodal culture, greenhouse acclimatization and genetic homogeneity assessment using molecular markers.

## Materials and Methods

### Plant material and culture preparation

**Fig 1:**
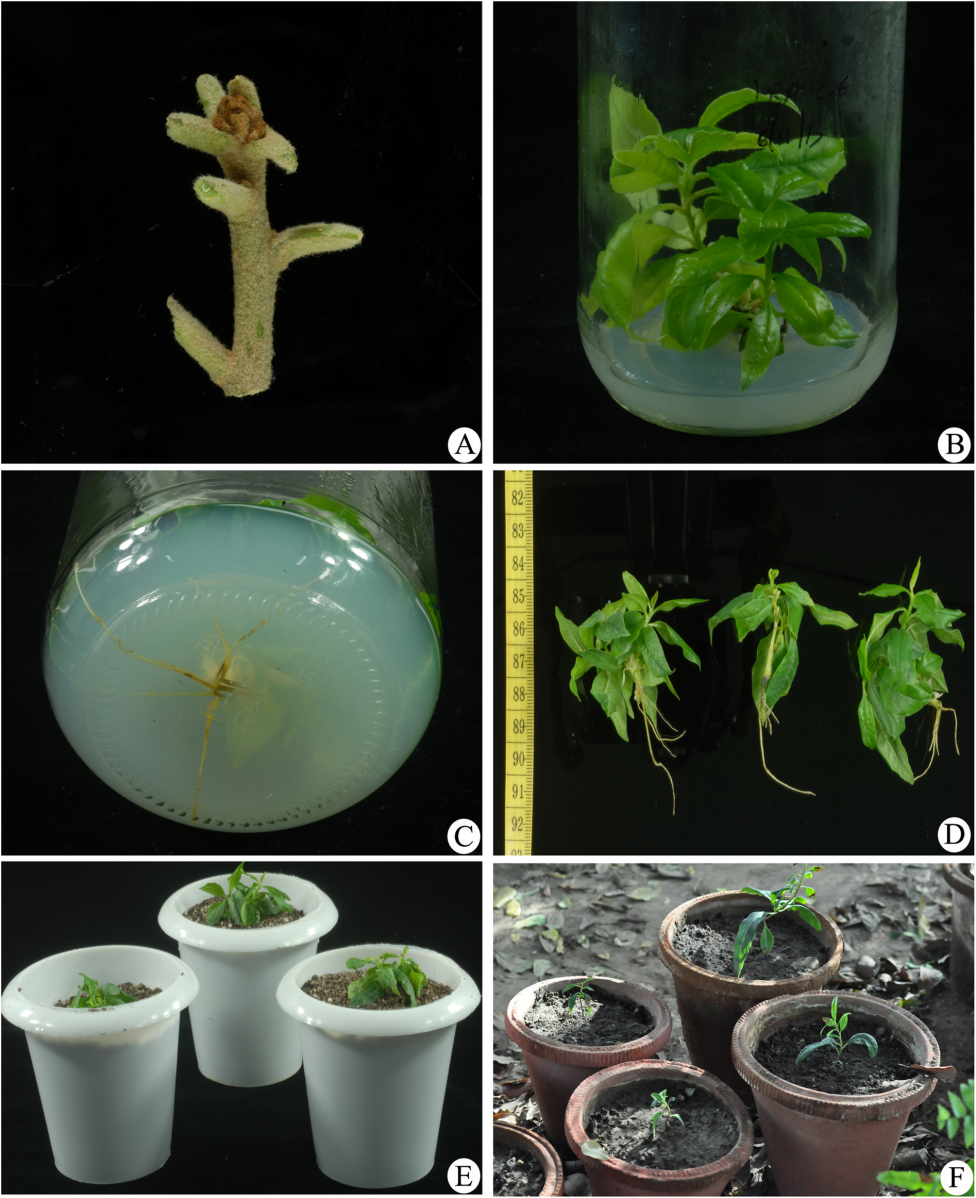
*Pittosporum eriocarpum*. (A) Nodal explant. (B) Shoot induction in MS medium supplemented with BA (mg/l). (C) Root induction in MS medium supplemented with IBA (mg/l). (D) *In vitro* grown plantlets. (E) *In vitro* grown plantlets in soil rite. (F) *In vitro* raised plantlets in natural conditions.

Healthy shoots were collected from three different wild populations from sexually mature plants growing in Dehradun (30^0^ 35.05’N, 78^0^ 01.97’E), Nainital (29^0^ 22.627’N, 79^0^ 28.163’E) and Jeullikot (29^0^ 27.144’N, 79^0^ 24.795’E) in Uttarakhand region during March-May. The field studies did not involve specific permission for these locations. Nodal regions (2cm long) were used as explants after removing all the leaves and explants were washed with 2% (v/v) tween-20 for 30 min under running tap water and then surface sterilized with 60% (v/v) ethyl alcohol for 10 minutes (Fig 1A). These explants were then soaked in 0.1% (w/v) mercuric chloride for 5 min followed by washing with autoclaved double distilled water under sterile conditions. Murashige and Skoog medium [15] supplemented with different growth hormones and 2% (w/v) sucrose 20gm/l and one set without growth regulators as a control were prepared. The medium was solidified with 0.8% (w/v) agar and pH was adjusted to 5.8 with 1N NaOH or 1N HCl before autoclaving at 121°C and 108 K Pa for 20 minutes. Cultures were incubated at 25±2°C, 55–60% RH, under 16 hour photoperiod with light intensity of 2000 lux provided by cool white fluorescent lights.

### Shoot induction and multiplication

To determine the role of various combinations of growth hormones on shooting, freshly excised stem explants were cultured vertically in full strength MS medium. We used different levels ofBAP and levels of IBA as factors. We assessed three effects i.e. BAP, IBA and the interaction between these two factors (BAPxIBA) on shoot length. There were 20 treatments to determine these three effects. Thus, the effect on shoot length were determined using 4x5 factorial design with BA varied at four levels (0.2, 0.4, 0.6, 0.8 mg/l) and IBA at five levels (0.0, 0.2, 0.4, 0.6, 0.8 mg/l), for a total of 20 treatments. Factorials were designed in a completely randomized manner. Each treatment combination, including the control had 25 replicates and the experiment was repeated twice. The average data from both experiments were combined for further statistical analysis. The data was analyzed by two-way ANOVA followed by Tukey’s multiple comparison test. Shoot length and number of shoots were recorded periodically. All cultures were maintained under the same growth conditions stated above and regenerated shoots were subcultured at least three times. After five months, regenerated shoots were separated and cut end of each was dipped for an hour in IBA and then subcultured in best suited combinations and change in the numbers of shoots, numbers of leaves per branch and callus diameter were recorded. One-way ANOVA was used to determine the effects of PGR combinations used for shoot multiplication on number of shoots, number of leaves per branch and callus diameter

### Rooting, acclimatization and field transfer

To determine the influence of various root hormones on root induction, multiple shoots produced in the shoot multiplication stage were separated carefully under sterile condition and subcultured in MS medium. We used three types of hormones (IAA, IBA and NAA) and different level of concentrations (0.2, 0.4, 0.6, 0.8, 1.0 mg/l) of each hormone as factors. The interaction between the types and concentrations of hormones was also analyzed. A 3x5 factorial design was used to determine the effects of PGRs (three) and their concentrations (Five) on number of roots per shoot, root length and basal callus formation separately. Two-way ANOVA was used followed by Tukey’s multiple comparison tests for statistical test. Replicates were handled in same manner as described in shoot and multiplication section. The cultures were maintained at 25±2°C, 55–60% RH under 16 hour photoperiod with light intensity of 2000 lux provided by cool white fluorescent lights.

Rooted shoots with minimum 2–3 roots were carefully harvested from culture flasks and washed thoroughly under running tap water to remove traces of agar. These plantlets were then planted in plastic pots (9 cm in diameter and 11 cm in height) containing autoclaved mixture of soil and sand in the ratio of 1:1. We covered the plantlets with a polythene cap for one month and small slits were made at the edges to maintain high relative humidity (RH 80±5%) and irrigated them every alternate day with half strength MS liquid medium and incubated in a culture room.

Micropropagated plantlets of minimum 5cm in height, after certain level of acclimatization were transferred on soil in culture room. Gradually rooted plantlets were transferred to greenhouse conditions (temperature 25°C and RH 45%) to allow them to acclimatize for 10 days, and gradual loosening of polythene caps was done and finally the caps were removed after 15 days.

After 3 months of exposition under greenhouse conditions, the micropropagation-derived plants were transferred to the nursery. The number of surviving plants was recorded periodically.

### Statistical analysis

All experiments were carried out in a completely randomized manner. The results were statistically analyzed using two-way Analysis of Variance (ANOVA) (Tables 1 and 2) and one-way ANOVA (Table 3) and through Statistical Packages for the Social Sciences (SPSS) version 16.0. Mean separation was doneusing Post-Hoc Tukey’s test for all the data (p<0.05) and presented in Tables 2 and 4. [16].

**Table 1:** Effect of various concentrations of BA and IBA (mg/l) on shoot length in *P*. *eriocarpum*.

BAP (mg/l)	IBA (mg/l)	Shoot length (mm)(Mean± SD)
**0.2**	00	28.14±1.4
**0.2**	0.2	26.03±5.7
**0.2**	0.4	27.71±0.1
**0.2**	0.6	25.59±0.4
**0.2**	0.8	26.98±0.1
**0.4**	0.0	27.96±0.7
**0.4**	0.2	27.38±0.1
**0.4**	0.4	30.98±0.6
**0.4**	0.6	43.34±3.8
**0.4**	0.8	31.95±1.1
**0.6**	0.0	30.27±0.1
**0.6**	0.2	31.61±1.16
**0.6**	0.4	33.44±0.2
**0.6**	0.6	31.37±1.1
**0.6**	0.8	27.51±0.8
**0.8**	0.0	28.72±0.8
**0.8**	0.2	28.32±0.7
**0.8**	0.4	28.08±0.4
**0.8**	0.6	28.21±0.3
**0.8**	0.8	26.41±0.8
**0.0**	0.0	0

**Table 2:** Effect of auxins (IAA, IBA or NAA) on *in vitro* rooting of *P*. *eriocarpum* shoots grown on MS medium.

Auxin	Concentration(mg/l)	Number of rootsper shoot±SD	Root length±SD(mm)	Presence of basal callus±SD(mm)
**IAA**	0.2	2.65±0.5	10.03±1.1	0
	0.4	6.00±0.8	13.05±2.9	0
	0.6	3.93±0.8	7.0±1.0	3.21±0.8
	0.8	3.53±1.1	5.56±1.1	5.57±0.4
	1.0	0	0	17.43±2.1
**IBA**	0.2	2.00±0.0	15.57±1.0	0
	0.4	2.55±0.5	18.29±3.9	0
	0.6	12.78±2.6	36.40±10.0	0
	0.8	2.03±0.8	7.68±1.3	0
	1.0	1.75±0.5	10.43±2.3	7.06±1.5
**NAA**	0.2	1.63±0.5	18.83±8.6	0
	0.4	7.63±0.5	22.47±3.1	7.18±1.5
	0.6	1.80±1.0	8.25±1.7	10.18±1.7
	0.8	1.5±0.6	13.16±1.4	12.06±0.7
	1.0	0	0	21.90±0.4
**Control**	0	0	0	0

**Table 3:** ANOVA of the effect of BA and IBA and combined BA and IBA on shoot length.

Source	Sum of squares	df	Mean Square	F-Value	p-Value
**BAP**	380	3	126	44_(3,60)_	<0.0001
**IBA**	172	4	43	15_(4,60)_	<0.0001
**BAPxIBA**	603	12	50	17_(12,60)_	<0.0001
**Pure Error**	173	60			
**Total (Corrected)**	1327	79			
**R-Squared**	0.87				
**Adj R-Squared**	0.83				

**Table 4:** ANOVA of the effect of growth hormones on number of roots/shoot, root length and basal callus formation.

Factors	Sum of squares	df	Mean Square	F	P-value
**No. of roots/shoot**					
**A. Hormone Type**	30	2	15	18_(2,45)_	<0.0001
**B. Concentration**	268	4	67	82_(4,45)_	<0.0001
**AxB**	314	8	39	48_(8,45)_	<0.0001
**Error**	37	45	0.9		
**Total (Corrected)**	649	59			
**R-Squared**	0.94				
**Adj R-Squared**	0.92				
**Root Length**					
**A. Hormone Type**	1112	2	556	36_(2,45)_	<0.0001
**B. Concentration**	1826	4	456	29_(4,45)_	<0.0001
**AxB**	1848	8	231	15_(8,45)_	<0.0001
**Error**	697	45	15		
**Total (Corrected)**	5484	59			
**R-Squared**	0.87				
**Adj R-Squared**	0.83				
**Basal Callus**					
**A. Hormone Type**	788	2	394	460_(2,45)_	<0.0001
**B. Concentration**	1683	4	421	491_(4,45)_	<0.0001
**AxB**	321	8	40	47_(8,45)_	<0.0001
**Error**	39	45	0.9		
**Total (Corrected)**	2830	59			
**R-Squared**	0.99				
**Adj R-Squared**	0.98				

### DNA isolation and genetic homogeneity assessment

To evaluate the genetic homogeneity of micropropagated plantlets, nine hardened plants were randomly selected along with the mother plant for genetic homogeneity assessment. Total genomic DNA of all plants was extracted from leaves using DNeasy Plant Minikit (Qiagen, Netherlands). The leaf material (25mg) was powdered in liquid nitrogen and stored at -80°C until extraction. The quality and quantity of DNA samples were estimated on 1.5% agarose gel and spectrophotometer respectively. The extracted DNA was further preceded for genetic homogeneity assessment by PCR based SCoT, ISSR and RAPD markers.

### SCoT analysis

Ten SCoT primers [17] were selected out of 20 primers screened for genetic homogeneity assessment. The PCR amplification was carried out in a total volume of 25 μl containing 2.5 μl of 10X PCR buffer containing 15 mM MgCl_2_, 0.2mM dNTPs, 1 unit Taq Polymerase (Sigma, USA), 20ng of genomic DNA and 20 ng of Primer (Integrated DNA technologies Inc., India). PCR was performed using following reaction conditions: one cycle of DNA denaturation at 94°C for 4 min, 38 cycles of 30 s denaturation at 94°C, annealing at Ta°C and 1 min of extension at 72°C with a final extension at 72°C for 8 min.

### ISSR analysis

After preliminary screening of 15 ISSR primers, 10 primers were selected. DNA amplification reaction was performed in a total volume of 25 μl containing 2.5 μl of 10X PCR buffer, 15 mM MgCl_2_, 0.2mM dNTPs, 1 unit Taq Polymerase (Sigma, USA), 20ng of genomic DNA and 20 ng of ISSR Primer (Sigma Aldrich, USA). PCR was performed using the following program: Initial denaturation at 94°C for 4 min, followed by 35 cycles of denaturation at 94°C for 40 s, annealing at Ta°C for 60 s and extension at 72°C for 60 s with a final extension at 72°C for 8 min.

### RAPD Analysis

RAPD analysis was done to support the results of SCoT and ISSR markers. A set of 10 decamer RAPD primers out of 15 primers (OPL 1–10) (Sigma Aldrich, USA) were used for amplification. The PCR amplification was carried out in a total volume of 25 μl containing 2.5 μl of 10X PCR buffer, 15 mM MgCl_2_, 0.2mM dNTPs, 1 unit Taq Polymerase (Sigma, USA), 20ng of genomic DNA and 20 ng of RAPD primer were subjected to one cycle of DNA denaturation at 94°C for 4 min, 45 cycles of 1 min denaturation at 94°C, 1 min annealing at Ta°C and 2 min of extension at 72°C with a final extension at 72°C for 7 min.

The annealing temperature (Ta°C) was kept at 2°C below the melting temperature of all the three primers used. We used AB Verity Thermal Cycler (USA) for amplification and repeated all the reactions twice. Amplified fragments were run on a 2.0% (w/v) agarose gel in 1X Tris acetate EDTA (TAE) Buffer stained by ethidium bromide (0.5 μg/ml). A 1 Kb ladder was used to estimate the size of unknown DNA fragments. Gels were documented using an UVP Gel Documentation (Jena, Germany).

### DataAnalysis

Only the consistent reproducible bands were scored and data matrix was prepared based on presence (1) and absence (0). Intensity of the band was not considered while scoring. NTSYSpc (Numerical Taxonomy and Multivariate Analysis System) Version 2.1 software [18] was used to perform the similarity matrix and construction of dendrogram. Genetic association amongst the different individuals was measured by the Jaccard’s similarity coefficient [19] with the SIMQUAL (Similarity for qualitative data program in NTSYS) module of NTSYS-pc software. The similarity matrix was subjected to cluster analysis of UPGMA and a dendrogram was generated by using the SAHN (sequential, agglomerative hierarchical and nested clustering) module in NTSYSpc program.

## Results and Discussion

### Shoot induction and multiplication

**Fig 2:**
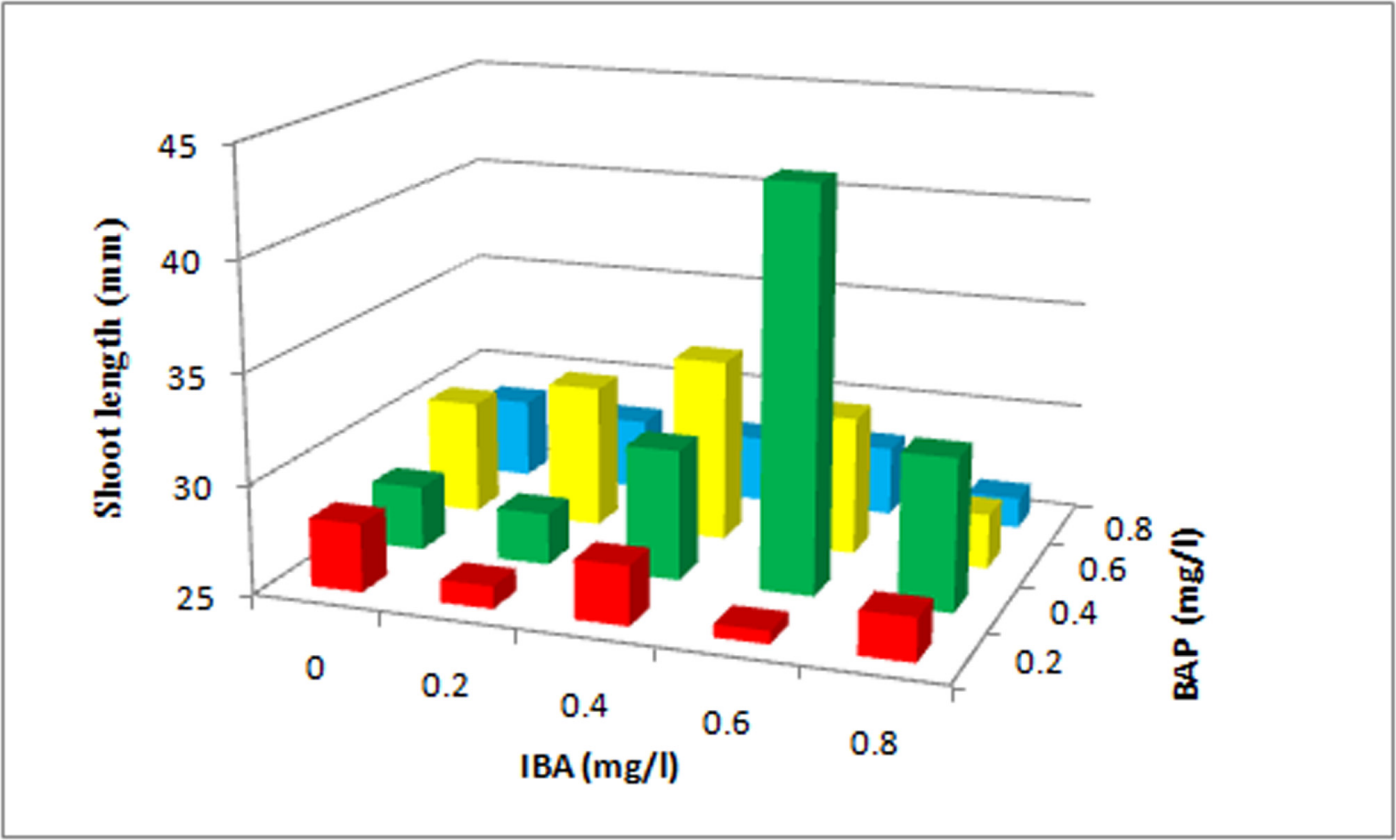
Effect of various concentrations of BA and IBA on shoot induction from nodal explants of *P*. *eriocarpum*.

**Fig 3:**
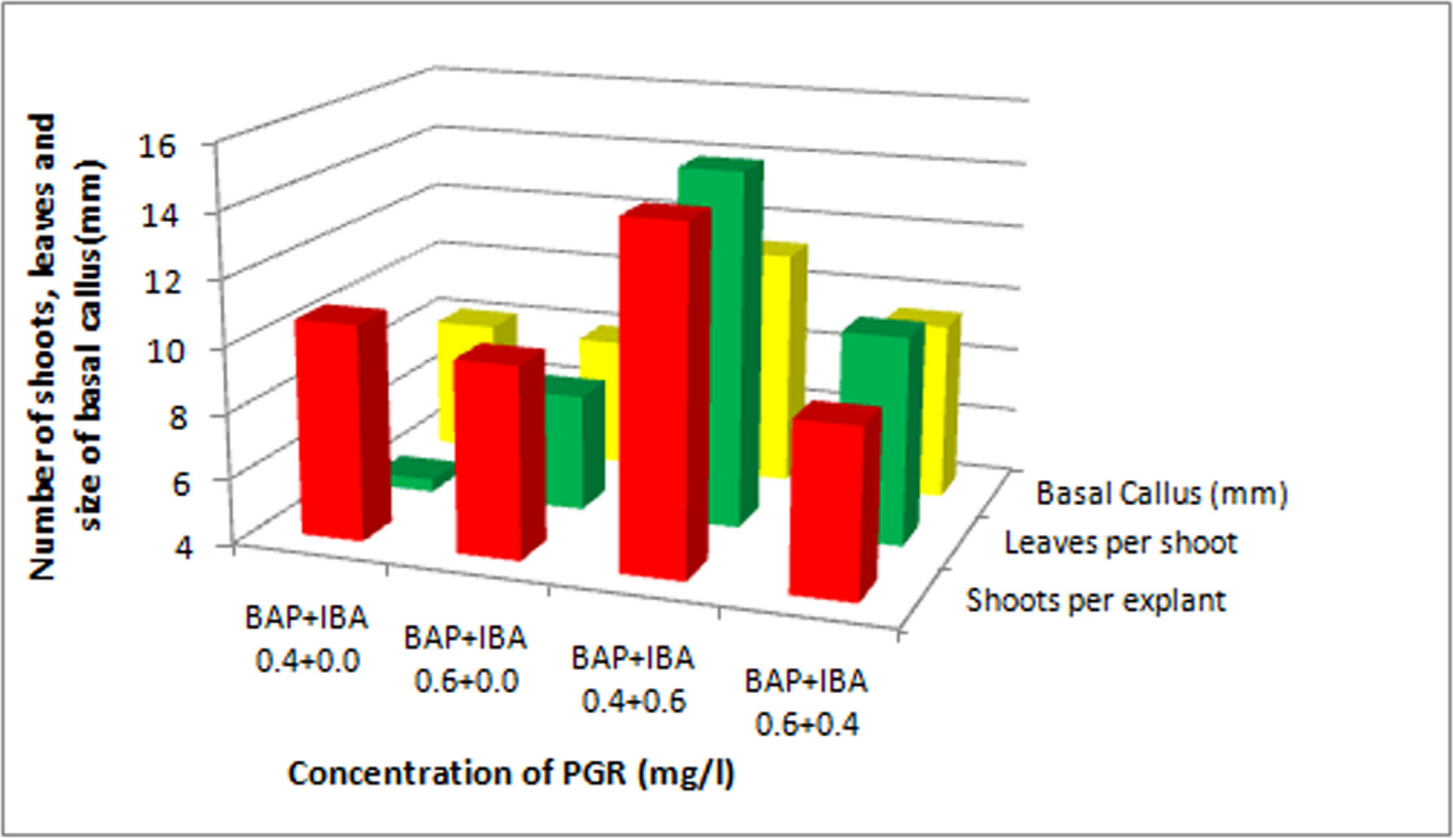
The effect of BA and IBA on number of shoots produced per explant, number of leaves per shoot and basal callus.

Explants were inoculated on MS medium with various concentrations of BA alone and in combination with IBA. A two-way ANOVA was conducted to determine the effects of BA, IBA and BA+IBA on shoot length. First shoot initiation was observed in BA 0.4mg/l after 8 days of culture. Among various concentrations of BA and IBA used, MS medium containing BAxIBA (0.4x0.6 mg/l) was found most suitable for shoot induction (93.3%), with 43.34±3.8 mm shoot length (Figs 1A–1B and 2) followed by BAxIBA (0.6x0.4 mg/l) which showed 86.7% shoot induction with 33.44±0.2 mm shoot length per explant (Table 1). Shoot induction frequency was significantly influenced by BA and IBA independently as well as their interaction was also found significant. The mean separation analysis by Tukey’s tests on shoot length showed that BAP has the largest effect on shoot length followed by interaction between BAPxIBA than IBA alone (Table 3). Out of 20 treatments at individuallevel, BAP in combination with IBA showed higher shoot regeneration frequency than BAP alone. BA is equivalent to 6-benzylaminopurine, BAP [20]. It is relevant to mention here that, the shoot induction is enhanced with the synergistic effect of cytokinin and auxin [21]. In the highest tested concentrations of BA (0.8 mg/l) alone and in combination with IBA (BA+IBA 0.8x0.2 mg/l), the shootlength was decreased. The explants of control set failed to develop shoots. The shoots obtained from BA 0.4 mg/l, BA 0.6 mg/l, BA+IBA 0.4+0.6 mg/l, BA+IBA 0.6+0.4 mg/l were further subcultured minimum of three times, and then after dipping in IBA for an hour were inoculated on MS medium in respective concentrations. Within five months of culture they developed multiple shoots. A one-way ANOVA was conducted to compare the effect of dipping of shoots obtained from four combinations on number of shoots per explant, leaves per branch and basal calls formation. BA+IBA 0.4+0.6 mg/l was found to be most suitable for multiplication of shoots with 14.40±3.3 shoots per explants and 14.85±1.7leaves per branch (Fig 3; Table 5). We observed significant increment in shoot multiplication after subculturing the mother plant. This may be due to suppression of apical dominance due to repeated subcultures that in turn induce basal meristematic cells to produce new shoots [22]. This method of shoot multiplication has been employed in other plant species [23]. Treatment of low level of auxin and cytokinin in culture medium is known to stimulate number and elongation of shoots in several tree species [24]. Regenerated shoots remained viable for more than eight months when left in original culture medium. This is a low cost and short term storage method for germplasm of *P*. *eriocarpum*. Since it is a medicinally important and endangered plant, use of this method will provide protocol for extraction of medicinal compound and release the pressure on natural populations.

**Table 5:** Effect of one hour incubation in IBA on number of shoots, leaves and callus diameter per explants.

PGR combinations used for shoot multiplication mg/l)	Number of shoots per explant±SD	Number of leaves per branch±SD	Callus diameter (mm)±SD
**BA 0.4**	10.60±1.1	4.39±0.5	8.11±0.6
**BA 0.6**	9.85±1.1	7.58±1.3	7.97±0.3
**BA 0.4+IBA0.6**	14.40±3.3	14.85±1.7	11.27±1.7
**BA0.6 +IBA0.4**	9.10±1.2	10.33±1.6	9.43±1.9

### Rooting, acclimatization and field transfer

**Fig 4:**
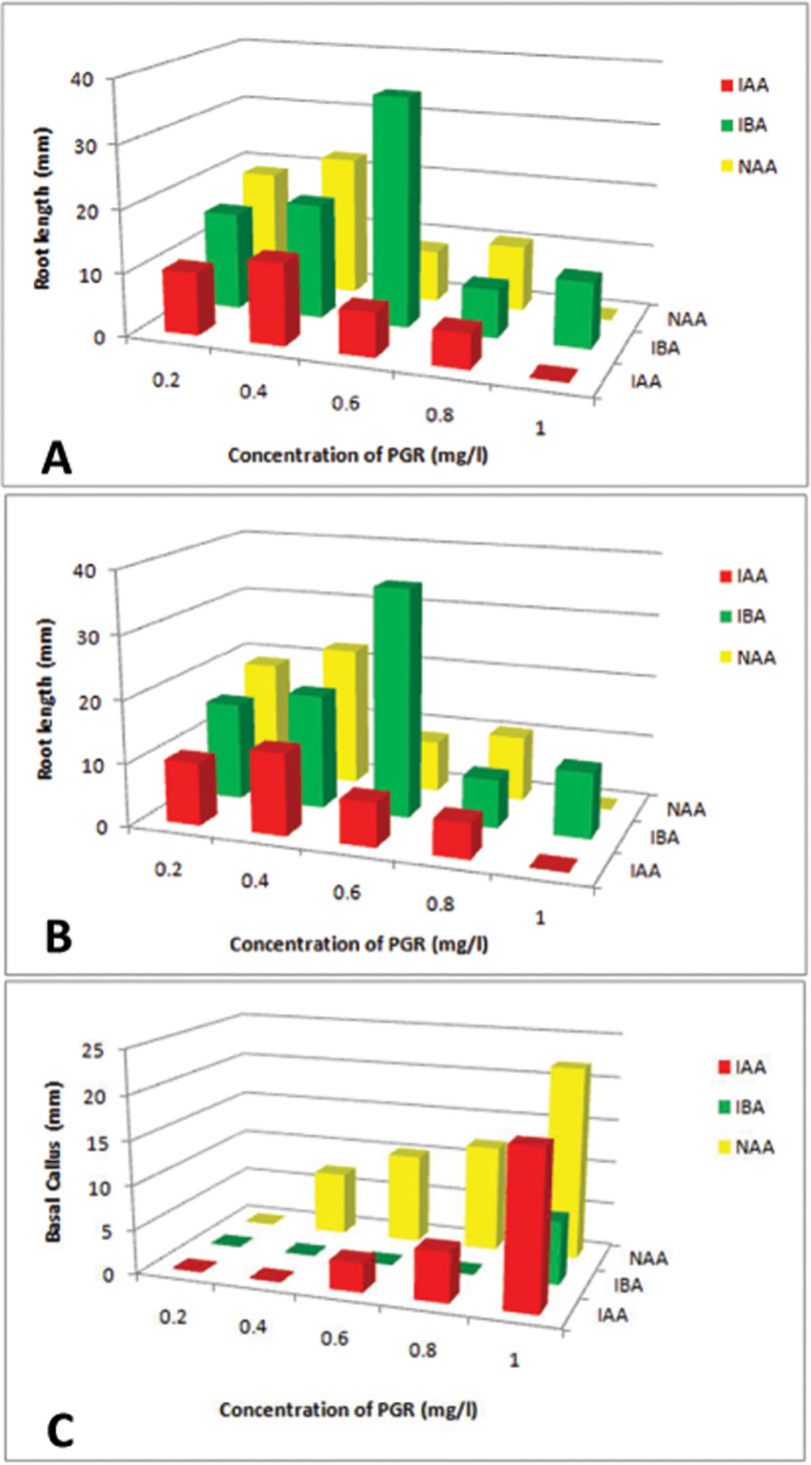
Effect of types of auxins on root induction in *P*. *eriocarpum*. (A)Number of roots produced per regenerated shoot. (B)Root length. (C) Basal callus formation per explant.

Strength of MS medium, concentration and type of auxin and supplementation of additives are a few factors which affect the rate of *in vitro* rooting [25]. *In vitro* raised shoots obtained from cultures on MS medium supplemented with BA alone and BA in combination with IBA were rooted in different types and concentrations of auxins (IAA, IBA, NAA). A two-way ANOVA was conducted to determine the effects of type of hormones at varied concentrations and interaction between the two on number of roots per explant, root length and basal callus formation. First rooting was observed in IBA 0.6 mg/l after 21 days of culture which showed 90% root induction with 12.78±2.6roots per shoot, 36.40±10.0 mm root length per root without any basal callus formation. It has been observed that with increase in the concentration of IBA, there was decrease in percentage of root induction, number of roots per shoot but varied in root length per root (Fig 1C, Table 2). IAA (0.4 mg/l) showed 80% rooting response with6.00±0.8roots per explant, 13.05±2.9 mm root length per root without any basal callus formation. Decrease in percentage of root induction, number of roots per explant and root length per root was observed with increase in IAA concentration. Among different concentrations of NAA tested, NAA 0.4mg/l showed highest rooting response (80%) with 7.63±0.5 roots per explant, 22.47±3.1 mm root length per root with basal callus formation. Increase in the concentration of NAA led to decrease in percentage of root induction and number of roots per explant, however, increase in basal callus formation was observed. All three effects, type ofhormones, hormone concentrations and the interactions between the two factors (hormone type x concentration) showed significant influence on root induction, root length and basal callusformation (Table 4). No rooting was observed in IAA and NAA at concentration 1.00 mg/l. Among the three PGRs, IBA (0.6mg/l) stimulated early initiation of roots followed by IAA (0.8 mg/l) and NAA (0.4mg/l). Also, IBA 0.6 mg/l showed maximum root induction, root numbers per shoot and length among three auxins tested. Nearly 80–85% rooted explants developed into plantlets (Fig 1D). Cultures raised in IBA 0.6 mg/l showed maximum survival rate (90%) followed by IAA and then NAA (Fig 4A–4C). IBA stimulated rooting has also been reported in *Pittosporum napaulensis*, where direct root organogenesis is observed in the medium supplemented with IBA and IAA, whereas higher concentration of NAA, led to the formation of callus [8]. IBA stimulated rooting is also reported in *Pittosporum tobira variegatum*[7]. Superiority of IBA as rooting hormone over other auxins is also reported in other genera [23, 26, 27].

Transfer of *in vitro* raised plantlets to a similar environment that exists in nature is likely to be most suitable for the growth and survival of plantlets [28]. As *P*. *eriocarpum* grows on exposed sites with temperature range of 12−27^0^ C,*in vitro* raised rooted plantlets were transferred to the greenhouse (temperature 25°C) for three months. Nearly 73% plantlets survived and continued to grow (Fig 1E and 1F). In many other taxa, high mortality rate has been reported for *in vitro* raised plants when transferred to natural filed conditions, as they possess weak root system at this stage [29,30]. In the present study, no phenotypic variation was found amongst the *in vitro* raised plants. The reintroduction of native plants and especially of rare and endangered species has become important in conservation worldwide for restoration purposes, which has also become an important practice in biodiversity conservation [31–35]. Since *P*. *eriocarpum* is native to India and endangered species, the reintroduction in nature would save the species from becoming extinct in nature.

### Genetic homogeneity assessment

We evaluated the genetic homogeneity of the *in vitro* grown plants through SCoT, ISSR and RAPD markers. The use of more than one marker has always been recommended for a better analysis of genetic homogeneity of plants because micropropagation is known to provoke somaclonal variation in micropropagated plants [36]. The RAPD and ISSR analyses are based on the non-coding regions of DNA used for studying genetic diversity, variability or stability. However, SCoT is novel and gene targeted molecular marker technique derived from flanking ATG translation codon in plant gene and is considered to be more authentic in assessing genetic homogeneity [17,37]. All these markers are found to be simple, resolvable, and cost-effective in assessing the genetic homogeneity [13, 38–40].

**Fig 5:**
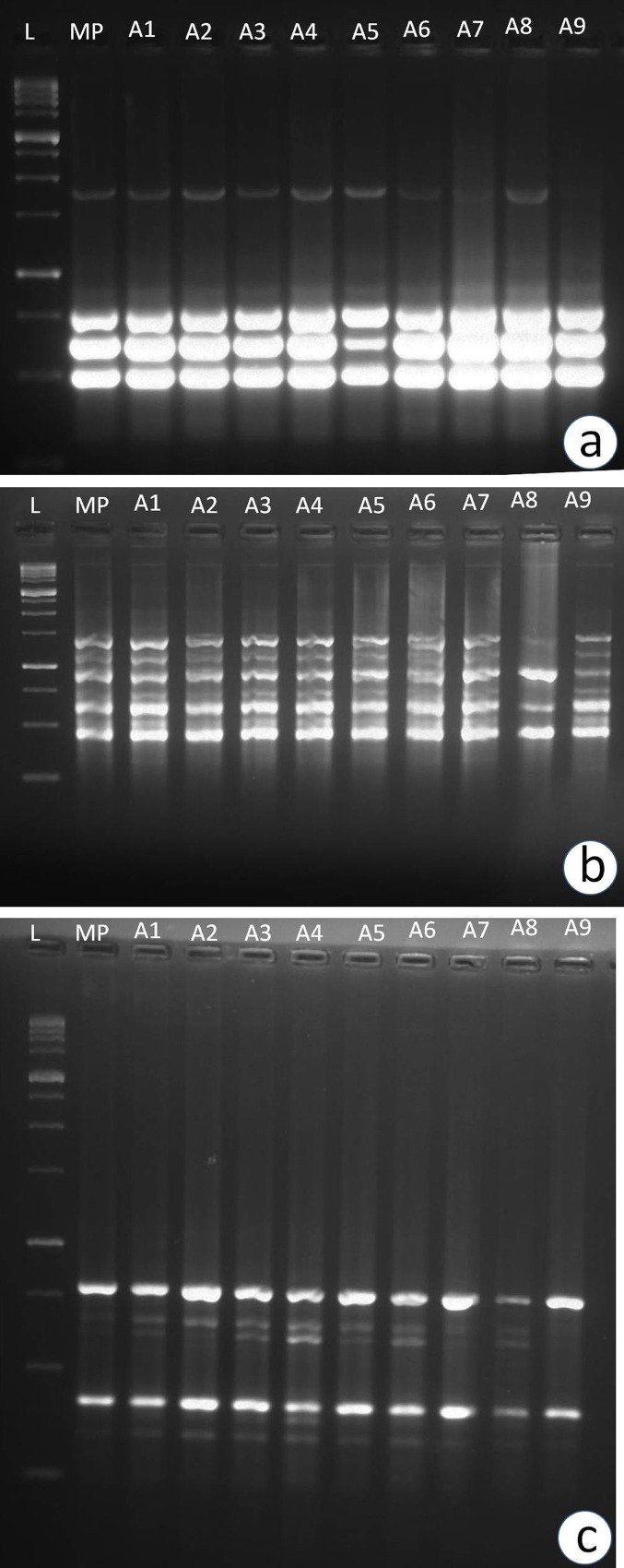
Assessment of genetic fidelity of *in vitro* raised plants of *Pittosporum eriocarpum*. (A) SCoT fragments obtained with primer SCoT 35. (B) ISSR fragments obtained from primer number 860. (C)RAPD fragments obtained with primer OPL10. L- 1Kb ladder; MP- mother plant (Control); A1-A9- *in vitro* raised plants.

Ten SCoT primers produced a total of 47 reproducible and scorable bands with an average of 4.7 bands per primer. Each primer produced amplification product in the range of 250–1500 bp. All banding profiles from micropropagated plants were monomophic and similar to mother plant except primers viz., primer 15 and 18, which showed polymorphic bands in two of the regenerates. Number of scorable band varied from 2 to 7 for each primer. In case of ISSR primers, 10 primers yielded reproducible bands out of 15 primers screened. They yielded 34 scorable bands with an average of 3.4 bands per primer ranging from 250–1500 bp. The band numbers varied from 2 to 7 in each primer. All banding profiles from micropropagated plants were monomophic and similar to mother plant except the primers viz., primer 847 and 860 which showed polymorphic bands in two of the regenerates. Of the 15 RAPD decamer primers screened initially, only 10 primers showed clear and reproducible bands. These primers gave rise to a total of 44 scorable bands. The number of bands for each primer varied from 1–7 with an average of 4.4 bands per primer ranging from 250 to 1000 bp. The highest number of bands were obtained in the primer 2 i.e., 7 and the lowest number of bands i.e., one, was obtained in the primer 9. All banding profiles from micropropagated plants were monomophic and similar to mother plant except the primer viz., primer OPL 10 which showed polymorphic bands in three of the regenerates. A comparative account of primer sequences used for SCoT, ISSR and RAPD analysis is given in Table 6. Monomorphic banding pattern was observed for all the amplified band classes from SCoT, ISSR and RAPD among the regenerates and mother plant, indicating the absence of variability (Fig 5A–5C).

**Table 6:** Amplification products generated with SCoT, ISSR and RAPD markers among mother plant and *in vitro* raised plants.

Markers	Primerseries	Primer sequence	Number of scorable bandsper primer	Monomorphic bands	Polymorphic bands
**SCoT**	1	5’CAACAATGGCTACCACCA3’	5	5	0
	2	5’CAACAATGGCTACCACCC3’	3	3	0
	3	5’CAACAATGGCTACCACCG3’	6	6	0
	4	5’CAACAATGGCTACCACCG3’	4	4	0
	5	5’CAACAATGGCTACCACCT3’	4	4	0
	10	5’CAACAATGGCTACCAGCC3’	3	3	0
	15	5’ACGACATGGCGACCGCGA3’	6	3	3
	18	5’ACCATGGCTACCACCGCC3’	7	3	4
	35	5’CATGGCTACCACCGGCCC3’	5	5	0
	36	5’GCAACAATGGCTACCACC3’	4	4	0
**ISSR**	843	5’CTCTCTCTCTCTCTCTRA3’	3	3	0
	847	5’CACACACACACACACARC3’	4	2	2
	852	5’TCTCTCTCTCTCTCTCRA3’	3	3	0
	853	5’TCTCTCTCTCTCTCTCRT3’	3	3	0
	854	5’TCTCTCTCTCTCTCTCRG3’	2	2	0
	855	5’ACACACACACACACYT3’	3	3	0
	857	5’ACACACACACACACYG3’	3	3	0
	860	5’TGTGTGTGTGTGTGTGRA3’	7	4	2
	888	5’BDBCACACACACACACA3’	4	4	0
	891	5’HVHTGTGTGTGTGTGTG3’	2	2	0
**RAPD**	OPL-01	5’GGCATGACCT3’	5	5	0
	OPL-02	5’TGGGCGTCAA3’	7	7	0
	OPL-03	5’CCAGCAGCTT3’	5	5	0
	OPL-04	5’GACTGCACAC3’	6	6	0
	OPL-05	5’ACGCAGGCAC3’	4	4	0
	OPL-06	5’GAGGGAAGAG3’	4	4	0
	OPL-07	5’AGGCGGGAAC3’	3	3	0
	OPL-08	5’AGCAGGTGGA3’	3	3	0
	OPL-09	5’TGCGAGAGTC3’	1	1	0
	OPL-10	5’TGGGAGATGG3’	6	4	2

### Data analysis

**Fig 6:**
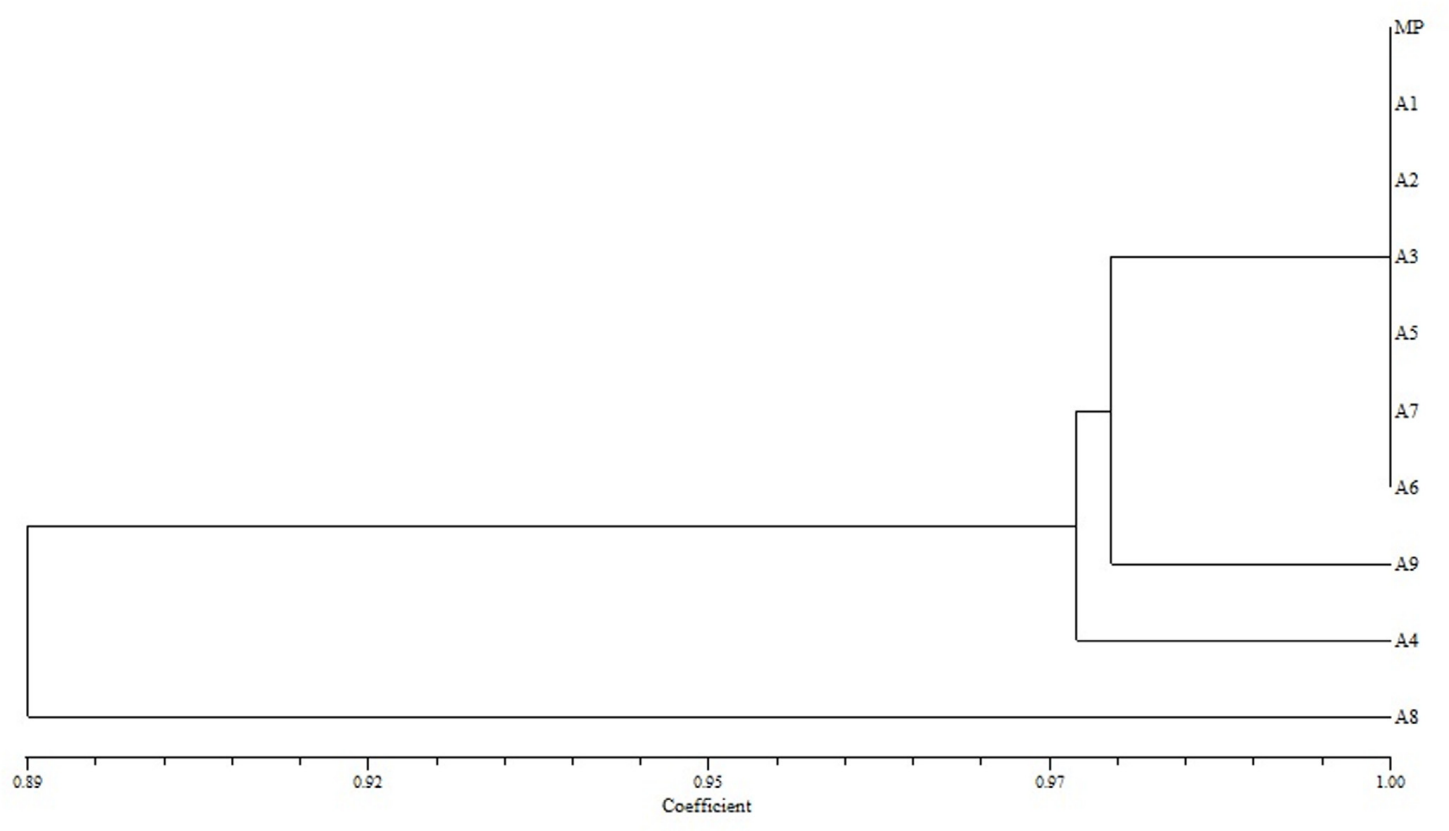
Dendrogram illustrating coefficient similarity among *in vitro* raised plants with mother plant of *P*. *eriocarpum* by UPGMA cluster analysis from the SCoT data set showing genetic relationship. MP- mother plant; A1-A9- *in vitro* raised plants.

**Fig 7:**
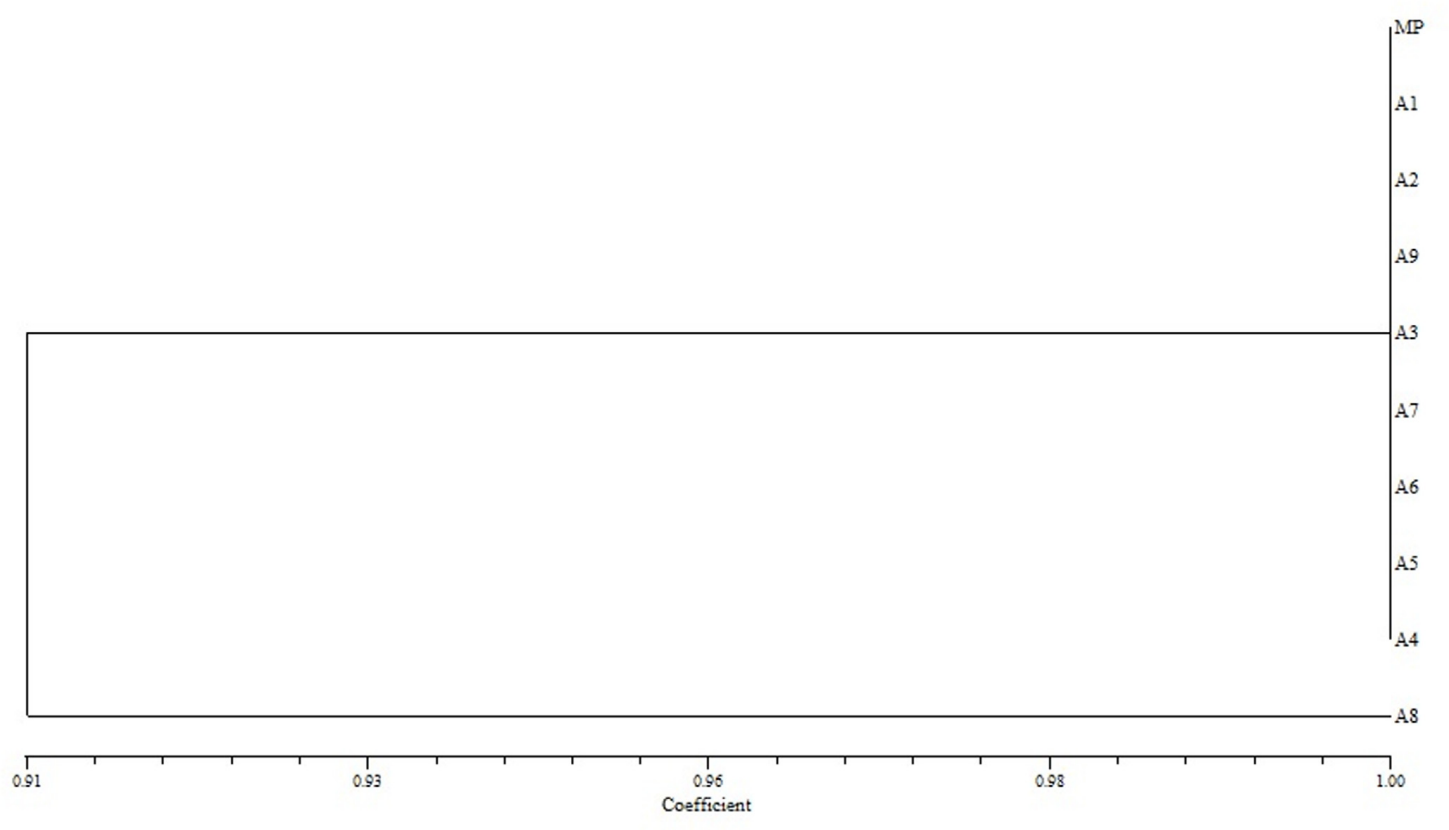
Dendrogram illustrating coefficient similarity among *in vitro* raised plants with mother plant of *P*. *eriocarpum* by UPGMA cluster analysis from the ISSR data set showing genetic relationship. MP- mother plant; A1-A9- *in vitro* raised plants.

**Fig 8:**
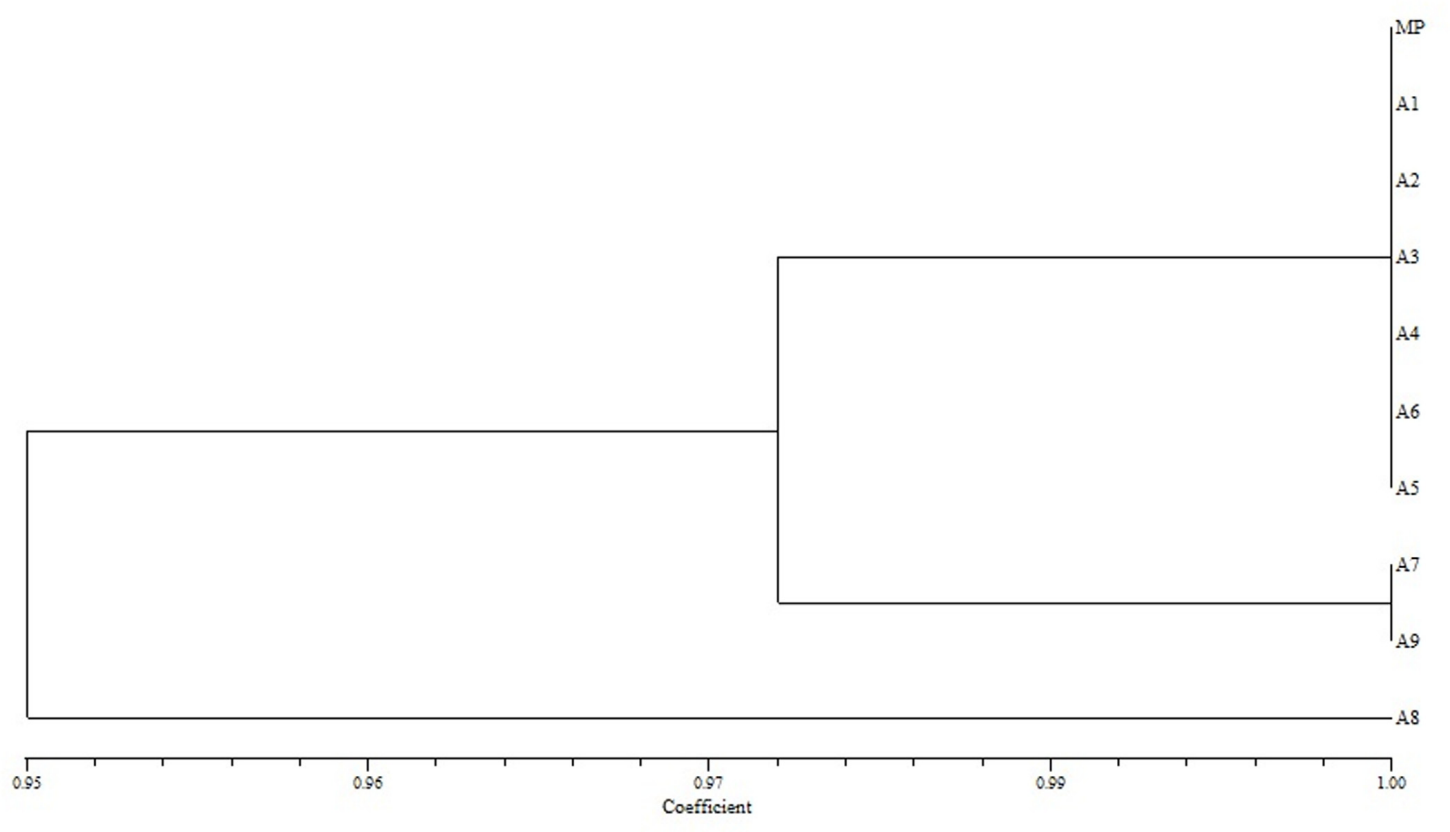
Dendrogram illustrating coefficient similarity among *in vitro* raised plants with mother plant of *P*. *eriocarpum* by UPGMA cluster analysis from the RAPD data set showing genetic relationship. MP- mother plant; A1-A9- *in vitro* raised plants.

The similarity value on the basis of Jaccard’s similarity coefficient among nine micropropagated plants and mother plant was calculated for three markers viz., SCoT, ISSR and RAPD which showed similarity ranged from 0.89 to 1.00, 0.91 to 1.00 and 0.95 to1.00 respectively (Figs 6–8). The dendrograms generated through UPGMA analysis revealed 97% similarity among regenerated plants with mother plant. Dendrogram generated for SCoT marker showed that A1, A2, A3, A5, A7 and A8 are very identical to mother plant. A9 and A4 also clustered close to MP. Dendrogram generated for ISSR marker showed that except A8, rest of the micropropagated plants were 100% identical to MP. Dendrogram generated for RAPD marker showed that A7 and A8 are almost identical to MP and A1-A6. It has been observed that, dendrograms generated for the three markers showed a slight change at DNA level of *in vitro* raised A8 plant which probably is due to somaclonal variation [11]. The dendrogram generated through UPGMA analysis revealed 97% similarity amongst the regenerates and the donor mother plant.

## Conclusions

Present study reports a holistic and reliable procedure for *in vitro* regeneration, greenhouse acclimatization and field transfer of *P*. *eriocarpum*. This is the first successful attempt to establish a micropropagation protocol for this species by nodal explants, which will provide a alternative method for its conservation as well as supply of the material for commercial use. It is estimated that a single explant can finally develop into 8–10 hardened plants within 8 months of culture period. We reported the genetic homogeneity and true-to-type nature of *in vitro* raised plants using SCoT, ISSR and RAPD analysis. Such protocol is useful for providing valuable resource material and help in the delisting of species from red data list.

## Supporting Information

S1 Table Effect of various concentrations of BA and IBA (mg/l) on shoot length in *P*. *eriocarpum*. (PDF)

S2 Table Effect of auxins (IAA, IBA or NAA) on *in vitro* rooting of *P*. *eriocarpum* shoots grown on MS medium. (PDF)

S3 Table ANOVA of the effect of BA and IBA and combined BA and IBA on shoot length (PDF)
