# Supplementary material for: Genetic Homogeneity Revealed Using SCoT, ISSR and RAPD Markers in Micropropagated Pittosporum eriocarpum Royle- An Endemic and Endangered Medicinal Plant
Source: PLoS One. 2016 Jul 19;11(7):e0159050. doi: 10.1371/journal.pone.0159050 (PMC4951010; doi:10.1371/journal.pone.0159050)
Supplement: S1 Table — (PDF) [file pone.0159050.s001.pdf]

```
UNIANOVA SL BY BAP IBA
  /METHOD=SSTYPE(3)
  /INTERCEPT=INCLUDE
  /EMMEANS=TABLES(OVERALL)
  /PRINT=HOMOGENEITY DESCRIPTIVE
  /CRITERIA=ALPHA(.05)
  /DESIGN=BAP IBA BAP*IBA.
```

Univariate Analysis of Variance

Notes

Output Created		29-MAY-2016 23:06:40
Comments		
Input	Data	C: \Users\SAMTA\Desktop\Julie\Table 1data file_1.sav
	Active Dataset	DataSet2
	Filter	<none>
	Weight	<none>
	Split File	<none>
	N of Rows in Working Data File	120
Missing Value Handling	Definition of Missing	User-defined missing values are treated as missing.
	Cases Used	Statistics are based on all cases with valid data for all variables in the model.
Syntax		UNIANOVA SL BY BAP IBA /METHOD=SSTYPE(3) /INTERCEPT=INCLUDE /EMMEANS=TABLES(OVERALL) /PRINT=HOMOGENEITY DESCRIPTIVE /CRITERIA=ALPHA(.05) /DESIGN=BAP IBA BAP*IBA.
Resources	Processor Time	00:00:00.03
	Elapsed Time	00:00:00.17

```
[DataSet2] C:\Users\SAMTA\Desktop\Julie\Table 1data file_1.sav
```

Between-Subjects Factors

		Value Label	N
BAP	1.00	0.2	20
	2.00	0.4	20
	3.00	0.6	20
	4.00	0.8	20
IBA	1.00	0.0	16
	2.00	0.2	16
	3.00	0.4	16
	4.00	0.6	16
	5.00	0.8	16

Descriptive Statistics

Dependent Variable: SL

BAP	IBA	Mean	Std. Deviation	N
0.2	0.0	28.1425	1.38911	4
	0.2	26.0300	5.75335	4
	0.4	27.7125	.11843	4
	0.6	25.5875	.35846	4
	0.8	26.9775	.11206	4
	Total	26.8900	2.55789	20
0.4	0.0	27.9550	.73278	4
	0.2	27.3775	.06238	4
	0.4	30.9775	.56512	4
	0.6	43.3375	3.84612	4
	0.8	31.9500	1.13502	4
	Total	32.3195	6.14752	20
0.6	0.0	30.2725	.13200	4
	0.2	31.6050	1.16360	4
	0.4	33.4400	.22405	4
	0.6	31.3700	1.11633	4
	0.8	27.5100	.80701	4
	Total	30.8395	2.12882	20
0.8	0.0	28.7150	.77328	4
	0.2	28.3225	.73277	4
	0.4	28.0750	.39846	4
	0.6	28.2050	.27477	4
	0.8	26.4150	.83036	4
	Total	27.9465	.99523	20
Total	0.0	28.7713	1.22529	16
	0.2	28.3338	3.39292	16
	0.4	30.0513	2.42892	16

Descriptive Statistics

Dependent Variable: SL

BAP	IBA	Mean	Std. Deviation	N
	0.6	32.1250	7.24021	16
	0.8	28.2131	2.37766	16
	Total	29.4989	4.09796	80

Levene's Test of Equality of Error Variances[a]

Dependent Variable: SL

F	df1	df2	Sig.
5.902	19	60	.000

Tests the null hypothesis that the error variance of the dependent variable is equal across groups.

a. Design: Intercept + BAP + IBA + BAP * IBA

Tests of Between-Subjects Effects

Dependent Variable: SL

Source	Type III Sum of Squares	df	Mean Square	F	Sig.
Corrected Model	1154.158[a]	19	60.745	21.127	.000
Intercept	69614.690	1	69614.690	24211.886	.000
BAP	379.386	3	126.462	43.983	.000
IBA	171.868	4	42.967	14.944	.000
BAP * IBA	602.904	12	50.242	17.474	.000
Error	172.514	60	2.875		
Total	70941.362	80			
Corrected Total	1326.671	79			

a. R Squared = .870 (Adjusted R Squared = .829)

Estimated Marginal Means

Grand Mean

Dependent Variable: SL

Mean	Std. Error	95% Confidence Interval	
		Lower Bound	Upper Bound
29.499	.190	29.120	29.878
