# Supplementary material for: Genetic Homogeneity Revealed Using SCoT, ISSR and RAPD Markers in Micropropagated Pittosporum eriocarpum Royle- An Endemic and Endangered Medicinal Plant
Source: PLoS One. 2016 Jul 19;11(7):e0159050. doi: 10.1371/journal.pone.0159050 (PMC4951010; doi:10.1371/journal.pone.0159050)
Supplement: S2 Table — (PDF) [file pone.0159050.s002.pdf]

```
ONEWAY ShootperExplant BY PGRs
  /STATISTICS DESCRIPTIVES HOMOGENEITY
  /MISSING ANALYSIS
  /POSTHOC=TUKEY ALPHA(0.05).
```

Oneway

Notes

Output Created		30-MAY-2016 00:19:59
Comments		
Input	Data	F: \4thApril_2wayAnova\Multiplication_rawFile_4thapril.sav
	Active Dataset	DataSet1
	Filter	<none>
	Weight	<none>
	Split File	<none>
	N of Rows in Working Data File	27
Missing Value Handling	Definition of Missing	User-defined missing values are treated as missing.
	Cases Used	Statistics for each analysis are based on cases with no missing data for any variable in the analysis.
Syntax		ONEWAY ShootperExplant BY PGRs /STATISTICS DESCRIPTIVES HOMOGENEITY /MISSING ANALYSIS..
Resources	Processor Time	00:00:00.03
	Elapsed Time	00:00:00.42

```
[DataSet1] F:\4thApril_2wayAnova\Multiplication_rawFile_4thapril.sav
```

Descriptives

ShootperExplant

	N	Mean	Std. Deviation	Std. Error	95% Confidence Interval for Mean	
					Lower Bound	Upper Bound
0.4BA	4	10.6000	1.14310	.57155	8.7811	12.4189
0.6BA	4	9.8500	1.07548	.53774	8.1387	11.5613
0.4BA 0.6IBA	4	14.4000	3.30252	1.65126	9.1449	19.6551
0.6BA 0.4IBA	4	9.1000	1.22746	.61373	7.1468	11.0532
Total	16	10.9875	2.72320	.68080	9.5364	12.4386

Descriptives

ShootperExplant

	Minimum	Maximum
0.4BA	9.40	12.00
0.6BA	8.40	11.00
0.4BA 0.6IBA	9.60	17.00
0.6BA 0.4IBA	7.40	10.00
Total	7.40	17.00

Test of Homogeneity of Variances

ShootperExplant

Levene Statistic	df1	df2	Sig.
2.265	3	12	.133

ANOVA

ShootperExplant

	Sum of Squares	df	Mean Square	F	Sig.
Between Groups	66.608	3	22.203	5.970	.010
Within Groups	44.630	12	3.719		
Total	111.238	15			

Post Hoc Tests

Multiple Comparisons

Dependent Variable: ShootperExplant

Tukey HSD

(I) PGRs	(J) PGRs	Mean Difference (I-J)	Std. Error	Sig.	95% Confidence Interval	
					Lower Bound	Upper Bound
0.4BA	0.6BA	.75000	1.36367	.945	-3.2986	4.7986
	0.4BA 0.6IBA	-3.80000	1.36367	.068	-7.8486	.2486
	0.6BA 0.4IBA	1.50000	1.36367	.696	-2.5486	5.5486
0.6BA	0.4BA	-.75000	1.36367	.945	-4.7986	3.2986
	0.4BA 0.6IBA	-4.55000*	1.36367	.026	-8.5986	-.5014
	0.6BA 0.4IBA	.75000	1.36367	.945	-3.2986	4.7986
0.4BA 0.6IBA	0.4BA	3.80000	1.36367	.068	-.2486	7.8486
	0.6BA	4.55000*	1.36367	.026	.5014	8.5986
	0.6BA 0.4IBA	5.30000*	1.36367	.010	1.2514	9.3486
0.6BA 0.4IBA	0.4BA	-1.50000	1.36367	.696	-5.5486	2.5486
	0.6BA	-.75000	1.36367	.945	-4.7986	3.2986
	0.4BA 0.6IBA	-5.30000*	1.36367	.010	-9.3486	-1.2514

*. The mean difference is significant at the 0.05 level.

Homogeneous Subsets

ShootperExplant

Tukey HSD[a]

PGRs	N	Subset for alpha = 0.05	
		1	2
0.6BA 0.4IBA	4	9.1000	
0.6BA	4	9.8500	
0.4BA	4	10.6000	10.6000
0.4BA 0.6IBA	4		14.4000
Sig.		.696	.068

Means for groups in homogeneous subsets are displayed.

a. Uses Harmonic Mean Sample Size = 4.000.

```
ONEWAY LeavesPerBranch BY PGRs
 /STATISTICS DESCRIPTIVES HOMOGENEITY
 /MISSING ANALYSIS
 /POSTHOC=TUKEY ALPHA(0.05).
```

Oneway

Notes

Output Created		30-MAY-2016 00:22:09
Comments		
Input	Data	F: \4thApril_2wayAnova\Multiplication_rawFile_4thapril.sav
	Active Dataset	DataSet1
	Filter	<none>
	Weight	<none>
	Split File	<none>
	N of Rows in Working Data File	27
Missing Value Handling	Definition of Missing	User-defined missing values are treated as missing.
	Cases Used	Statistics for each analysis are based on cases with no missing data for any variable in the analysis.
Syntax		ONEWAY LeavesPerBranch BY PGRs<br>/STATISTICS DESCRIPTIVES HOMOGENEITY<br>/MISSING ANALYSIS<br>/POSTHOC=TUKEY ALPHA(0.05).
Resources	Processor Time	00:00:00.03
	Elapsed Time	00:00:00.34

```
[DataSet1] F:\4thApril_2wayAnova\Multiplication_rawFile_4thapril.sav
```

Descriptives

LeavesPerBranch

	N	Mean	Std. Deviation	Std. Error	95% Confidence Interval for Mean	
					Lower Bound	Upper Bound
0.4BA	4	4.3900	.48484	.24242	3.6185	5.1615
0.6BA	4	7.5750	1.26062	.63031	5.5691	9.5809
0.4BA 0.6IBA	4	14.8500	1.70000	.85000	12.1449	17.5551
0.6BA 0.4IBA	4	10.3300	1.56916	.78458	7.8331	12.8269
Total	16	9.2863	4.14178	1.03544	7.0793	11.4932

Descriptives

LeavesPerBranch

	Minimum	Maximum
0.4BA	4.00	5.00
0.6BA	6.00	9.00
0.4BA 0.6IBA	12.40	16.00
0.6BA 0.4IBA	8.32	12.00
Total	4.00	16.00

Test of Homogeneity of Variances

LeavesPerBranch

Levene Statistic	df1	df2	Sig.
1.159	3	12	.366

ANOVA

LeavesPerBranch

	Sum of Squares	df	Mean Square	F	Sig.
Between Groups	235.785	3	78.595	43.807	.000
Within Groups	21.530	12	1.794		
Total	257.315	15			

Post Hoc Tests

Multiple Comparisons

Dependent Variable: LeavesPerBranch

Tukey HSD

(I) PGRs	(J) PGRs	Mean Difference (I-J)	Std. Error	Sig.	95% Confidence Interval	
					Lower Bound	Upper Bound
0.4BA	0.6BA	-3.18500*	.94713	.025	-5.9969	-.3731
	0.4BA 0.6IBA	-10.46000*	.94713	.000	-13.2719	-7.6481
	0.6BA 0.4IBA	-5.94000*	.94713	.000	-8.7519	-3.1281
0.6BA	0.4BA	3.18500*	.94713	.025	.3731	5.9969
	0.4BA 0.6IBA	-7.27500*	.94713	.000	-10.0869	-4.4631
	0.6BA 0.4IBA	-2.75500	.94713	.055	-5.5669	.0569
0.4BA 0.6IBA	0.4BA	10.46000*	.94713	.000	7.6481	13.2719
	0.6BA	7.27500*	.94713	.000	4.4631	10.0869
	0.6BA 0.4IBA	4.52000*	.94713	.002	1.7081	7.3319
0.6BA 0.4IBA	0.4BA	5.94000*	.94713	.000	3.1281	8.7519
	0.6BA	2.75500	.94713	.055	-.0569	5.5669
	0.4BA 0.6IBA	-4.52000*	.94713	.002	-7.3319	-1.7081

*. The mean difference is significant at the 0.05 level.

Homogeneous Subsets

LeavesPerBranch

Tukey HSD[a]

PGRs	N	Subset for alpha = 0.05		
		1	2	3
0.4BA	4	4.3900		
0.6BA	4		7.5750	
0.6BA 0.4IBA	4		10.3300	
0.4BA 0.6IBA	4			14.8500
Sig.		1.000	.055	1.000

Means for groups in homogeneous subsets are displayed.

a. Uses Harmonic Mean Sample Size = 4.000.

```
ONEWAY CallusDiameter BY PGRs
 /STATISTICS DESCRIPTIVES HOMOGENEITY
 /MISSING ANALYSIS
 /POSTHOC=TUKEY ALPHA(0.05).
```

Oneway

Notes

Output Created		30-MAY-2016 00:22:54
Comments		
Input	Data	F:<br>\4thApril_2wayAnova\Multiplication_rawFile_4thapril.sav
	Active Dataset	DataSet1
	Filter	<none>
	Weight	<none>
	Split File	<none>
	N of Rows in Working Data File	27
Missing Value Handling	Definition of Missing	User-defined missing values are treated as missing.
	Cases Used	Statistics for each analysis are based on cases with no missing data for any variable in the analysis.
Syntax		ONEWAY CallusDiameter BY PGRs<br>/STATISTICS DESCRIPTIVES HOMOGENEITY<br>/MISSING ANALYSIS<br>/POSTHOC=TUKEY ALPHA(0.05).
Resources	Processor Time	00:00:00.03
	Elapsed Time	00:00:00.08

```
[DataSet1] F:\4thApril_2wayAnova\Multiplication_rawFile_4thapril.sav
```

Descriptives

CallusDiameter

	N	Mean	Std. Deviation	Std. Error	95% Confidence Interval for Mean Lower Bound	95% Confidence Interval for Mean Upper Bound
0.4BA	4	8.1075	.63174	.31587	7.1023	9.1127
0.6BA	4	7.9650	.33191	.16596	7.4369	8.4931
0.4BA 0.6IBA	4	11.2675	1.69159	.84580	8.5758	13.9592
0.6BA 0.4IBA	4	9.4275	1.90783	.95392	6.3917	12.4633
Total	16	9.1919	1.81128	.45282	8.2267	10.1570

Descriptives

CallusDiameter

	Minimum	Maximum
0.4BA	7.39	8.93
0.6BA	7.51	8.28
0.4BA 0.6IBA	10.00	13.67
0.6BA 0.4IBA	8.03	12.14
Total	7.39	13.67

Test of Homogeneity of Variances

CallusDiameter

Levene Statistic	df1	df2	Sig.
2.149	3	12	.147

ANOVA

CallusDiameter

	Sum of Squares	df	Mean Square	F	Sig.
Between Groups	28.179	3	9.393	5.359	.014
Within Groups	21.032	12	1.753		
Total	49.211	15			

Post Hoc Tests

Multiple Comparisons

Dependent Variable: CallusDiameter

Tukey HSD

(I) PGRs	(J) PGRs	Mean Difference (I-J)	Std. Error	Sig.	95% Confidence Interval	
					Lower Bound	Upper Bound
0.4BA	0.6BA	.14250	.93612	.999	-2.6367	2.9217
	0.4BA 0.6IBA	-3.16000*	.93612	.025	-5.9392	-.3808
	0.6BA 0.4IBA	-1.32000	.93612	.517	-4.0992	1.4592
0.6BA	0.4BA	-.14250	.93612	.999	-2.9217	2.6367
	0.4BA 0.6IBA	-3.30250*	.93612	.019	-6.0817	-.5233
	0.6BA 0.4IBA	-1.46250	.93612	.434	-4.2417	1.3167
0.4BA 0.6IBA	0.4BA	3.16000*	.93612	.025	.3808	5.9392
	0.6BA	3.30250*	.93612	.019	.5233	6.0817
	0.6BA 0.4IBA	1.84000	.93612	.254	-.9392	4.6192
0.6BA 0.4IBA	0.4BA	1.32000	.93612	.517	-1.4592	4.0992
	0.6BA	1.46250	.93612	.434	-1.3167	4.2417
	0.4BA 0.6IBA	-1.84000	.93612	.254	-4.6192	.9392

*. The mean difference is significant at the 0.05 level.

Homogeneous Subsets

CallusDiameter

Tukey HSD[a]

PGRs	N	Subset for alpha = 0.05	
		1	2
0.6BA	4	7.9650	
0.4BA	4	8.1075	
0.6BA 0.4IBA	4	9.4275	9.4275
0.4BA 0.6IBA	4		11.2675
Sig.		.434	.254

Means for groups in homogeneous subsets are displayed.

a. Uses Harmonic Mean Sample Size = 4.000.
