# Supplementary material for: Genetic Homogeneity Revealed Using SCoT, ISSR and RAPD Markers in Micropropagated Pittosporum eriocarpum Royle- An Endemic and Endangered Medicinal Plant
Source: PLoS One. 2016 Jul 19;11(7):e0159050. doi: 10.1371/journal.pone.0159050 (PMC4951010; doi:10.1371/journal.pone.0159050)
Supplement: S3 Table — (PDF) [file pone.0159050.s003.pdf]

```
UNIANOVA RootPerShoot BY HormoneType Concentration
  /METHOD=SSTYPE(3)
  /INTERCEPT=INCLUDE
  /POSTHOC=HormoneType Concentration(TUKEY)
  /EMMEANS=TABLES(OVERALL)
  /EMMEANS=TABLES(HormoneType) COMPARE ADJ(LSD)
  /EMMEANS=TABLES(Concentration) COMPARE ADJ(LSD)
  /EMMEANS=TABLES(HormoneType*Concentration)
  /PRINT=HOMOGENEITY DESCRIPTIVE
  /CRITERIA=ALPHA(.05)
  /DESIGN=HormoneType Concentration HormoneType*Concentration.
```

Univariate Analysis of Variance

Notes

Output Created		29-MAY-2016 23:31:52
Comments		
Input	Data	C:\Users\SAMTA\Desktop\Julie\table 3 a,b,c12thApril twao way anova data file.sav
	Active Dataset	DataSet1
	Filter	<none>
	Weight	<none>
	Split File	<none>
	N of Rows in Working Data File	60
Missing Value Handling	Definition of Missing	User-defined missing values are treated as missing.
	Cases Used	Statistics are based on all cases with valid data for all variables in the model.

Notes

Syntax		UNIANOVA RootPerShoot BY HormoneType Concentration<br>/METHOD=SSTYPE(3)<br>/INTERCEPT=INCLUDE<br>/POSTHOC=HormoneType Concentration(TUKEY)<br>/EMMEANS=TABLES(OVERALL)<br>/EMMEANS=TABLES (HormoneType) COMPARE ADJ (LSD)<br>/EMMEANS=TABLES (Concentration) COMPARE ADJ (LSD)<br>/EMMEANS=TABLES (HormoneType*Concentration)<br>/PRINT=HOMOGENEITY DESCRIPTIVE<br>/CRITERIA=ALPHA(.05)<br>/DESIGN=HormoneType Concentration HormoneType*Concentration.
Resources	Processor Time	00:00:00.11
	Elapsed Time	00:00:00.64

```
[DataSet1] C:\Users\SAMTA\Desktop\Julie\table 3 a,b,c12thApril twao way anova
 data file.sav
```

Between-Subjects Factors

		Value Label	N
HormoneType	1.00	IAA	20
	2.00	IBA	20
	3.00	NAA	20
Concentration	1.00	0.2	12
	2.00	0.4	12
	3.00	0.6	12
	4.00	0.8	12
	5.00	1.0	12

Descriptive Statistics

Dependent Variable: RootPerShoot

HormoneType	Concentration	Mean	Std. Deviation	N
IAA	0.2	2.6500	.47258	4
	0.4	6.0000	.81650	4
	0.6	3.9250	.83016	4
	0.8	3.5250	1.05000	4
	1.0	.0000	.00000	4
	Total	3.2200	2.10378	20
IBA	0.2	2.0000	.00000	4
	0.4	2.5500	.52599	4
	0.6	12.7750	2.58247	4
	0.8	2.0250	.81803	4
	1.0	1.7500	.50000	4
	Total	4.2200	4.53578	20
NAA	0.2	1.6250	.47871	4
	0.4	7.6250	.47871	4
	0.6	1.8000	.97980	4
	0.8	1.5000	.57735	4
	1.0	.0000	.00000	4
	Total	2.5100	2.75622	20
Total	0.2	2.0917	.56481	12
	0.4	5.3917	2.28133	12
	0.6	6.1667	5.18746	12
	0.8	2.3500	1.17357	12
	1.0	.5833	.90034	12
	Total	3.3167	3.31622	60

Levene's Test of Equality of Error Variances[a]

Dependent Variable: RootPerShoot

F	df1	df2	Sig.
3.279	14	45	.001

Tests the null hypothesis that the error variance of the dependent variable is equal across groups.

a. Design: Intercept + HormoneType + Concentration + HormoneType * Concentration

Tests of Between-Subjects Effects

Dependent Variable: RootPerShoot

Source	Type III Sum of Squares	df	Mean Square	F	Sig.
Corrected Model	611.948[a]	14	43.711	53.313	.000
Intercept	660.017	1	660.017	805.007	.000
HormoneType	29.521	2	14.761	18.003	.000
Concentration	268.012	4	67.003	81.722	.000
HormoneType * Concentration	314.415	8	39.302	47.936	.000
Error	36.895	45	.820		
Total	1308.860	60			
Corrected Total	648.843	59			

a. R Squared = .943 (Adjusted R Squared = .925)

Estimated Marginal Means

1. Grand Mean

Dependent Variable: RootPerShoot

Mean	Std. Error	95% Confidence Interval	
		Lower Bound	Upper Bound
3.317	.117	3.081	3.552

2. HormoneType

Estimates

Dependent Variable: RootPerShoot

HormoneType	Mean	Std. Error	95% Confidence Interval	
			Lower Bound	Upper Bound
IAA	3.220	.202	2.812	3.628
IBA	4.220	.202	3.812	4.628
NAA	2.510	.202	2.102	2.918

Pairwise Comparisons

Dependent Variable: RootPerShoot

(I) HormoneType	(J) HormoneType	Mean Difference (I-J)	Std. Error	Sig.[b]	95% Confidence ...[b]
					Lower Bound
IAA	IBA	-1.000*	.286	.001	-1.577
	NAA	.710*	.286	.017	.133
IBA	IAA	1.000*	.286	.001	.423
	NAA	1.710*	.286	.000	1.133
NAA	IAA	-.710*	.286	.017	-1.287
	IBA	-1.710*	.286	.000	-2.287

Pairwise Comparisons

Dependent Variable: RootPerShoot

(I) HormoneType	(J) HormoneType	95% Confidence ...[b]
		Upper Bound
IAA	IBA	-.423
	NAA	1.287
IBA	IAA	1.577
	NAA	2.287
NAA	IAA	-.133
	IBA	-1.133

Based on estimated marginal means

*. The mean difference is significant at the .05 level.

b. Adjustment for multiple comparisons: Least Significant Difference (equivalent to no adjustments).

Univariate Tests

Dependent Variable: RootPerShoot

	Sum of Squares	df	Mean Square	F	Sig.
Contrast	29.521	2	14.761	18.003	.000
Error	36.895	45	.820		

The F tests the effect of HormoneType. This test is based on the linearly independent pairwise comparisons among the estimated marginal means.

3. Concentration

Estimates

Dependent Variable: RootPerShoot

Concentration	Mean	Std. Error	95% Confidence Interval	
			Lower Bound	Upper Bound
0.2	2.092	.261	1.565	2.618
0.4	5.392	.261	4.865	5.918
0.6	6.167	.261	5.640	6.693
0.8	2.350	.261	1.824	2.876
1.0	.583	.261	.057	1.110

Pairwise Comparisons

Dependent Variable: RootPerShoot

(I) Concentration	(J) Concentration	Mean Difference (I-J)	Std. Error	Sig.[b]	95% Confidence ...[b]
					Lower Bound
0.2	0.4	-3.300*	.370	.000	-4.045
	0.6	-4.075*	.370	.000	-4.820
	0.8	-.258	.370	.488	-1.003
	1.0	1.508*	.370	.000	.764
0.4	0.2	3.300*	.370	.000	2.555
	0.6	-.775*	.370	.042	-1.520
	0.8	3.042*	.370	.000	2.297
	1.0	4.808*	.370	.000	4.064
0.6	0.2	4.075*	.370	.000	3.330
	0.4	.775*	.370	.042	.030
	0.8	3.817*	.370	.000	3.072
	1.0	5.583*	.370	.000	4.839
0.8	0.2	.258	.370	.488	-.486
	0.4	-3.042*	.370	.000	-3.786
	0.6	-3.817*	.370	.000	-4.561
	1.0	1.767*	.370	.000	1.022
1.0	0.2	-1.508*	.370	.000	-2.253
	0.4	-4.808*	.370	.000	-5.553
	0.6	-5.583*	.370	.000	-6.328
	0.8	-1.767*	.370	.000	-2.511

Pairwise Comparisons

Dependent Variable: RootPerShoot

(I) Concentration	(J) Concentration	95% Confidence ...[b] Upper Bound
0.2	0.4	-2.555
	0.6	-3.330
	0.8	.486
	1.0	2.253
0.4	0.2	4.045
	0.6	-.030
	0.8	3.786
	1.0	5.553
0.6	0.2	4.820
	0.4	1.520
	0.8	4.561
	1.0	6.328
0.8	0.2	1.003
	0.4	-2.297
	0.6	-3.072
	1.0	2.511
1.0	0.2	-.764
	0.4	-4.064
	0.6	-4.839
	0.8	-1.022

Based on estimated marginal means

*. The mean difference is significant at the .05 level.

b. Adjustment for multiple comparisons: Least Significant Difference (equivalent to no adjustments).

Univariate Tests

Dependent Variable: RootPerShoot

	Sum of Squares	df	Mean Square	F	Sig.
Contrast	268.012	4	67.003	81.722	.000
Error	36.895	45	.820		

The F tests the effect of Concentration. This test is based on the linearly independent pairwise comparisons among the estimated marginal means.

4. HormoneType * Concentration

Dependent Variable: RootPerShoot

HormoneType	Concentration	Mean	Std. Error	95% Confidence Interval	
				Lower Bound	Upper Bound
IAA	0.2	2.650	.453	1.738	3.562
	0.4	6.000	.453	5.088	6.912
	0.6	3.925	.453	3.013	4.837
	0.8	3.525	.453	2.613	4.437
	1.0	1.025E-013	.453	-.912	.912
IBA	0.2	2.000	.453	1.088	2.912
	0.4	2.550	.453	1.638	3.462
	0.6	12.775	.453	11.863	13.687
	0.8	2.025	.453	1.113	2.937
	1.0	1.750	.453	.838	2.662
NAA	0.2	1.625	.453	.713	2.537
	0.4	7.625	.453	6.713	8.537
	0.6	1.800	.453	.888	2.712
	0.8	1.500	.453	.588	2.412
	1.0	-1.014E-013	.453	-.912	.912

Post Hoc Tests

HormoneType

Multiple Comparisons

Dependent Variable: RootPerShoot

Tukey HSD

(I) HormoneType	(J) HormoneType	Mean Difference (I-J)	Std. Error	Sig.	95% ...
					Lower Bound
IAA	IBA	-1.0000*	.28634	.003	-1.6940
	NAA	.7100*	.28634	.044	.0160
IBA	IAA	1.0000*	.28634	.003	.3060
	NAA	1.7100*	.28634	.000	1.0160
NAA	IAA	-.7100*	.28634	.044	-1.4040
	IBA	-1.7100*	.28634	.000	-2.4040

Multiple Comparisons

Dependent Variable: RootPerShoot

Tukey HSD

(I) HormoneType	(J) HormoneType	95% ... Upper Bound
IAA	IBA	-.3060
	NAA	1.4040
IBA	IAA	1.6940
	NAA	2.4040
NAA	IAA	-.0160
	IBA	-1.0160

Based on observed means.
The error term is Mean Square(Error) = .820.

*. The mean difference is significant at the .05 level.

Homogeneous Subsets

RootPerShoot

Tukey HSD[a,b]

HormoneType	N	Subset 1	2	3
NAA	20	2.5100		
IAA	20		3.2200	
IBA	20			4.2200
Sig.		1.000	1.000	1.000

Means for groups in homogeneous subsets are displayed.
Based on observed means.
The error term is Mean Square(Error) = .820.

a. Uses Harmonic Mean Sample Size = 20.000.

b. Alpha = .05.

Concentration

Multiple Comparisons

Dependent Variable: RootPerShoot

Tukey HSD

(I) Concentration	(J) Concentration	Mean Difference (I-J)	Std. Error	Sig.	95% ... Lower Bound
0.2	0.4	-3.3000*	.36966	.000	-4.3504
	0.6	-4.0750*	.36966	.000	-5.1254
	0.8	-.2583	.36966	.956	-1.3087
	1.0	1.5083*	.36966	.002	.4580
0.4	0.2	3.3000*	.36966	.000	2.2496
	0.6	-.7750	.36966	.239	-1.8254
	0.8	3.0417*	.36966	.000	1.9913
	1.0	4.8083*	.36966	.000	3.7580
0.6	0.2	4.0750*	.36966	.000	3.0246
	0.4	.7750	.36966	.239	-.2754
	0.8	3.8167*	.36966	.000	2.7663
	1.0	5.5833*	.36966	.000	4.5330
0.8	0.2	.2583	.36966	.956	-.7920
	0.4	-3.0417*	.36966	.000	-4.0920
	0.6	-3.8167*	.36966	.000	-4.8670
	1.0	1.7667*	.36966	.000	.7163
1.0	0.2	-1.5083*	.36966	.002	-2.5587
	0.4	-4.8083*	.36966	.000	-5.8587
	0.6	-5.5833*	.36966	.000	-6.6337
	0.8	-1.7667*	.36966	.000	-2.8170

Multiple Comparisons

Dependent Variable: RootPerShoot

Tukey HSD

(I) Concentration	(J) Concentration	95% ... Upper Bound
0.2	0.4	-2.2496
	0.6	-3.0246
	0.8	.7920
	1.0	2.5587
0.4	0.2	4.3504
	0.6	.2754
	0.8	4.0920
	1.0	5.8587
0.6	0.2	5.1254
	0.4	1.8254
	0.8	4.8670
	1.0	6.6337
0.8	0.2	1.3087
	0.4	-1.9913
	0.6	-2.7663
	1.0	2.8170
1.0	0.2	-.4580
	0.4	-3.7580
	0.6	-4.5330
	0.8	-.7163

Based on observed means.
The error term is Mean Square(Error) = .820.

*. The mean difference is significant at the .05 level.

Homogeneous Subsets

RootPerShoot

Tukey HSD[a,b]

Concentration	N	Subset		
		1	2	3
1.0	12	.5833		
0.2	12		2.0917	
0.8	12		2.3500	
0.4	12			5.3917
0.6	12			6.1667
Sig.		1.000	.956	.239

Means for groups in homogeneous subsets are displayed.
Based on observed means.
The error term is Mean Square(Error) = .820.

a. Uses Harmonic Mean Sample Size = 12.000.

b. Alpha = .05.

```
UNIANOVA RootLength BY HormoneType Concentration
 /METHOD=SSTYPE(3)
 /INTERCEPT=INCLUDE
 /POSTHOC=HormoneType Concentration(TUKEY)
 /EMMEANS=TABLES(OVERALL)
 /EMMEANS=TABLES(HormoneType) COMPARE ADJ(LSD)
 /EMMEANS=TABLES(Concentration) COMPARE ADJ(LSD)
 /EMMEANS=TABLES(HormoneType*Concentration)
 /PRINT=HOMOGENEITY DESCRIPTIVE
 /CRITERIA=ALPHA(.05)
 /DESIGN=HormoneType Concentration HormoneType*Concentration.
```

Univariate Analysis of Variance

Notes

Output Created		29-MAY-2016 23:33:49
Comments		
Input	Data	C:\Users\SAMTA\Desktop\Julie\table 3 a,b,c12thApril twao way anova data file.sav
	Active Dataset	DataSet1
	Filter	<none>
	Weight	<none>
	Split File	<none>
	N of Rows in Working Data File	60
Missing Value Handling	Definition of Missing	User-defined missing values are treated as missing.
	Cases Used	Statistics are based on all cases with valid data for all variables in the model.
Syntax		UNIANOVA RootLength BY HormoneType Concentration<br>/METHOD=SSTYPE(3)<br>/INTERCEPT=INCLUDE<br>/POSTHOC=HormoneType Concentration(TUKEY)<br>/EMMEANS=TABLES(OVERALL)<br>/EMMEANS=TABLES (HormoneType) COMPARE ADJ (LSD)<br>/EMMEANS=TABLES (Concentration) COMPARE ADJ (LSD)<br>/EMMEANS=TABLES (HormoneType*Concentration)<br>/PRINT=HOMOGENEITY DESCRIPTIVE<br>/CRITERIA=ALPHA(.05)<br>/DESIGN=HormoneType Concentration HormoneType*Concentration.
Resources	Processor Time	00:00:00.11
	Elapsed Time	00:00:00.33

```
[DataSet1] C:\Users\SAMTA\Desktop\Julie\table 3 a,b,c12thApril twao way anova
 data file.sav
```

Between-Subjects Factors

		Value Label	N
HormoneType	1.00	IAA	20
	2.00	IBA	20
	3.00	NAA	20
Concentration	1.00	0.2	12
	2.00	0.4	12
	3.00	0.6	12
	4.00	0.8	12
	5.00	1.0	12

Descriptive Statistics

Dependent Variable: RootLength

HormoneType	Concentration	Mean	Std. Deviation	N
IAA	0.2	10.0250	1.07308	4
	0.4	13.0475	2.96766	4
	0.6	6.9950	1.00785	4
	0.8	5.5625	1.08106	4
	1.0	.0000	.00000	4
	Total	7.1260	4.71988	20
IBA	0.2	15.5700	.97806	4
	0.4	18.2850	3.96607	4
	0.6	36.3950	10.36647	4
	0.8	7.6825	1.25985	4
	1.0	10.4275	2.32381	4
	Total	17.6720	11.29481	20
NAA	0.2	18.8325	8.63941	4
	0.4	22.4650	3.10810	4
	0.6	8.2450	1.71327	4
	0.8	13.1550	1.39117	4
	1.0	.0000	.00000	4
	Total	12.5395	8.95669	20
Total	0.2	14.8092	5.94566	12
	0.4	17.9325	5.05169	12
	0.6	17.2117	15.21175	12
	0.8	8.8000	3.52728	12
	1.0	3.4758	5.27562	12
	Total	12.4458	9.64086	60

Levene's Test of Equality of Error Variances[a]

Dependent Variable: RootLength

F	df1	df2	Sig.
4.564	14	45	.000

Tests the null hypothesis that the error variance of the dependent variable is equal across groups.

a. Design: Intercept + HormoneType + Concentration + HormoneType * Concentration

Tests of Between-Subjects Effects

Dependent Variable: RootLength

Source	Type III Sum of Squares	df	Mean Square	F	Sig.
Corrected Model	4786.467[a]	14	341.891	22.062	.000
Intercept	9293.926	1	9293.926	599.735	.000
HormoneType	1112.444	2	556.222	35.893	.000
Concentration	1825.860	4	456.465	29.456	.000
HormoneType * Concentration	1848.163	8	231.020	14.908	.000
Error	697.352	45	15.497		
Total	14777.746	60			
Corrected Total	5483.820	59			

a. R Squared = .873 (Adjusted R Squared = .833)

Estimated Marginal Means

1. Grand Mean

Dependent Variable: RootLength

Mean	Std. Error	95% Confidence Interval	
		Lower Bound	Upper Bound
12.446	.508	11.422	13.469

2. HormoneType

Estimates

Dependent Variable: RootLength

HormoneType	Mean	Std. Error	95% Confidence Interval	
			Lower Bound	Upper Bound
IAA	7.126	.880	5.353	8.899
IBA	17.672	.880	15.899	19.445
NAA	12.540	.880	10.767	14.312

Pairwise Comparisons

Dependent Variable: RootLength

(I) HormoneType	(J) HormoneType	Mean Difference (I-J)	Std. Error	Sig.[b]	95% Confidence ...[b]
					Lower Bound
IAA	IBA	-10.546*	1.245	.000	-13.053
	NAA	-5.414*	1.245	.000	-7.921
IBA	IAA	10.546*	1.245	.000	8.039
	NAA	5.133*	1.245	.000	2.625
NAA	IAA	5.414*	1.245	.000	2.906
	IBA	-5.133*	1.245	.000	-7.640

Pairwise Comparisons

Dependent Variable: RootLength

(I) HormoneType	(J) HormoneType	95% Confidence ...[b]
		Upper Bound
IAA	IBA	-8.039
	NAA	-2.906
IBA	IAA	13.053
	NAA	7.640
NAA	IAA	7.921
	IBA	-2.625

Based on estimated marginal means

*. The mean difference is significant at the .05 level.

b. Adjustment for multiple comparisons: Least Significant Difference (equivalent to no adjustments).

Univariate Tests

Dependent Variable: RootLength

	Sum of Squares	df	Mean Square	F	Sig.
Contrast	1112.444	2	556.222	35.893	.000
Error	697.352	45	15.497		

The F tests the effect of HormoneType. This test is based on the linearly independent pairwise comparisons among the estimated marginal means.

3. Concentration

Estimates

Dependent Variable: RootLength

Concentration	Mean	Std. Error	95% Confidence Interval	
			Lower Bound	Upper Bound
0.2	14.809	1.136	12.520	17.098
0.4	17.933	1.136	15.644	20.221
0.6	17.212	1.136	14.923	19.500
0.8	8.800	1.136	6.511	11.089
1.0	3.476	1.136	1.187	5.765

Pairwise Comparisons

Dependent Variable: RootLength

(I) Concentration	(J) Concentration	Mean Difference (I-J)	Std. Error	Sig.[b]	95% Confidence ...[b]
					Lower Bound
0.2	0.4	-3.123	1.607	.058	-6.360
	0.6	-2.403	1.607	.142	-5.639
	0.8	6.009*	1.607	.001	2.772
	1.0	11.333*	1.607	.000	8.096
0.4	0.2	3.123	1.607	.058	-.114
	0.6	.721	1.607	.656	-2.516
	0.8	9.133*	1.607	.000	5.896
	1.0	14.457*	1.607	.000	11.220
0.6	0.2	2.403	1.607	.142	-.834
	0.4	-.721	1.607	.656	-3.958
	0.8	8.412*	1.607	.000	5.175
	1.0	13.736*	1.607	.000	10.499
0.8	0.2	-6.009*	1.607	.001	-9.246
	0.4	-9.133*	1.607	.000	-12.369
	0.6	-8.412*	1.607	.000	-11.649
	1.0	5.324*	1.607	.002	2.087

Pairwise Comparisons

Dependent Variable: RootLength

(I) Concentration	(J) Concentration	95% Confidence ...[b] Upper Bound
0.2	0.4	.114
	0.6	.834
	0.8	9.246
	1.0	14.570
0.4	0.2	6.360
	0.6	3.958
	0.8	12.369
	1.0	17.694
0.6	0.2	5.639
	0.4	2.516
	0.8	11.649
	1.0	16.973
0.8	0.2	-2.772
	0.4	-5.896
	0.6	-5.175
	1.0	8.561

Pairwise Comparisons

Dependent Variable: RootLength

(I) Concentration	(J) Concentration	Mean Difference (I-J)	Std. Error	Sig.[b]	95% Confidence ...[b] Lower Bound
1.0	0.2	-11.333*	1.607	.000	-14.570
	0.4	-14.457*	1.607	.000	-17.694
	0.6	-13.736*	1.607	.000	-16.973
	0.8	-5.324*	1.607	.002	-8.561

Pairwise Comparisons

Dependent Variable: RootLength

(I) Concentration	(J) Concentration	95% Confidence ...[b] Upper Bound
1.0	0.2	-8.096
	0.4	-11.220
	0.6	-10.499
	0.8	-2.087

Based on estimated marginal means

*. The mean difference is significant at the .05 level.

b. Adjustment for multiple comparisons: Least Significant Difference (equivalent to no adjustments).

Univariate Tests

Dependent Variable: RootLength

	Sum of Squares	df	Mean Square	F	Sig.
Contrast	1825.860	4	456.465	29.456	.000
Error	697.352	45	15.497		

The F tests the effect of Concentration. This test is based on the linearly independent pairwise comparisons among the estimated marginal means.

4. HormoneType * Concentration

Dependent Variable: RootLength

HormoneType	Concentration	Mean	Std. Error	95% Confidence Interval	
				Lower Bound	Upper Bound
IAA	0.2	10.025	1.968	6.061	13.989
	0.4	13.048	1.968	9.083	17.012
	0.6	6.995	1.968	3.031	10.959
	0.8	5.563	1.968	1.598	9.527
	1.0	1.027E-013	1.968	-3.964	3.964
IBA	0.2	15.570	1.968	11.606	19.534
	0.4	18.285	1.968	14.321	22.249
	0.6	36.395	1.968	32.431	40.359
	0.8	7.683	1.968	3.718	11.647
	1.0	10.428	1.968	6.463	14.392
NAA	0.2	18.833	1.968	14.868	22.797
	0.4	22.465	1.968	18.501	26.429
	0.6	8.245	1.968	4.281	12.209
	0.8	13.155	1.968	9.191	17.119
	1.0	-1.346E-013	1.968	-3.964	3.964

Post Hoc Tests

HormoneType

Multiple Comparisons

Dependent Variable: RootLength

Tukey HSD

(I) HormoneType	(J) HormoneType	Mean Difference (I-J)	Std. Error	Sig.	95% ...
					Lower Bound
IAA	IBA	-10.5460*	1.24486	.000	-13.5631
	NAA	-5.4135*	1.24486	.000	-8.4306
IBA	IAA	10.5460*	1.24486	.000	7.5289
	NAA	5.1325*	1.24486	.000	2.1154
NAA	IAA	5.4135*	1.24486	.000	2.3964
	IBA	-5.1325*	1.24486	.000	-8.1496

Multiple Comparisons

Dependent Variable: RootLength

Tukey HSD

(I) HormoneType	(J) HormoneType	95% ...
		Upper Bound
IAA	IBA	-7.5289
	NAA	-2.3964
IBA	IAA	13.5631
	NAA	8.1496
NAA	IAA	8.4306
	IBA	-2.1154

Based on observed means.
The error term is Mean Square(Error) = 15.497.

*. The mean difference is significant at the .05 level.

Homogeneous Subsets

RootLength

Tukey HSD[a,b]

HormoneType	N	Subset		
		1	2	3
IAA	20	7.1260		
NAA	20		12.5395	
IBA	20			17.6720
Sig.		1.000	1.000	1.000

Means for groups in homogeneous subsets are displayed.
Based on observed means.
The error term is Mean Square(Error) = 15.497.

a. Uses Harmonic Mean Sample Size = 20.000.

b. Alpha = .05.

Concentration

Multiple Comparisons

Dependent Variable: RootLength

Tukey HSD

(I) Concentration	(J) Concentration	Mean Difference (I-J)	Std. Error	Sig.	95% ...
					Lower Bound
0.2	0.4	-3.1233	1.60710	.310	-7.6898
	0.6	-2.4025	1.60710	.571	-6.9690
	0.8	6.0092*	1.60710	.005	1.4427
	1.0	11.3333*	1.60710	.000	6.7668
0.4	0.2	3.1233	1.60710	.310	-1.4432
	0.6	.7208	1.60710	.991	-3.8457
	0.8	9.1325*	1.60710	.000	4.5660
	1.0	14.4567*	1.60710	.000	9.8902
0.6	0.2	2.4025	1.60710	.571	-2.1640
	0.4	-.7208	1.60710	.991	-5.2873
	0.8	8.4117*	1.60710	.000	3.8452
	1.0	13.7358*	1.60710	.000	9.1693
0.8	0.2	-6.0092*	1.60710	.005	-10.5757
	0.4	-9.1325*	1.60710	.000	-13.6990
	0.6	-8.4117*	1.60710	.000	-12.9782
	1.0	5.3242*	1.60710	.015	.7577
1.0	0.2	-11.3333*	1.60710	.000	-15.8998
	0.4	-14.4567*	1.60710	.000	-19.0232
	0.6	-13.7358*	1.60710	.000	-18.3023
	0.8	-5.3242*	1.60710	.015	-9.8907

Multiple Comparisons

Dependent Variable: RootLength

Tukey HSD

(I) Concentration	(J) Concentration	95% ... Upper Bound
0.2	0.4	1.4432
	0.6	2.1640
	0.8	10.5757
	1.0	15.8998
0.4	0.2	7.6898
	0.6	5.2873
	0.8	13.6990
	1.0	19.0232
0.6	0.2	6.9690
	0.4	3.8457
	0.8	12.9782
	1.0	18.3023
0.8	0.2	-1.4427
	0.4	-4.5660
	0.6	-3.8452
	1.0	9.8907
1.0	0.2	-6.7668
	0.4	-9.8902
	0.6	-9.1693
	0.8	-.7577

Based on observed means.
The error term is Mean Square(Error) = 15.497.

*. The mean difference is significant at the .05 level.

Homogeneous Subsets

RootLength

Tukey HSD[a,b]

Concentration	N	Subset 1	2	3
1.0	12	3.4758		
0.8	12		8.8000	
0.2	12			14.8092
0.6	12			17.2117
0.4	12			17.9325
Sig.		1.000	1.000	.310

Means for groups in homogeneous subsets are displayed.
Based on observed means.
The error term is Mean Square(Error) = 15.497.

a. Uses Harmonic Mean Sample Size = 12.000.

b. Alpha = .05.

```
UNIANOVA BasalCallus BY HormoneType Concentration
 /METHOD=SSTYPE(3)
 /INTERCEPT=INCLUDE
 /POSTHOC=HormoneType Concentration(TUKEY)
 /EMMEANS=TABLES(OVERALL)
 /EMMEANS=TABLES(HormoneType) COMPARE ADJ(LSD)
 /EMMEANS=TABLES(Concentration) COMPARE ADJ(LSD)
 /EMMEANS=TABLES(HormoneType*Concentration)
 /PRINT=HOMOGENEITY DESCRIPTIVE
 /CRITERIA=ALPHA(.05)
 /DESIGN=HormoneType Concentration HormoneType*Concentration.
```

Univariate Analysis of Variance

Notes

Output Created		29-MAY-2016 23:34:51
Comments		
Input	Data	C:\Users\SAMTA\Desktop\Julie\table 3 a,b,c12thApril twao way anova data file.sav
	Active Dataset	DataSet1
	Filter	<none>
	Weight	<none>
	Split File	<none>
	N of Rows in Working Data File	60
Missing Value Handling	Definition of Missing	User-defined missing values are treated as missing.
	Cases Used	Statistics are based on all cases with valid data for all variables in the model.
Syntax		UNIANOVA BasalCallus BY HormoneType Concentration<br>/METHOD=SSTYPE(3)<br>/INTERCEPT=INCLUDE<br>/POSTHOC=HormoneType Concentration(TUKEY)<br>/EMMEANS=TABLES(OVERALL)<br>/EMMEANS=TABLES (HormoneType) COMPARE ADJ (LSD)<br>/EMMEANS=TABLES (Concentration) COMPARE ADJ (LSD)<br>/EMMEANS=TABLES (HormoneType*Concentration)<br>/PRINT=HOMOGENEITY DESCRIPTIVE<br>/CRITERIA=ALPHA(.05)<br>/DESIGN=HormoneType Concentration HormoneType*Concentration.
Resources	Processor Time	00:00:00.11
	Elapsed Time	00:00:00.17

```
[DataSet1] C:\Users\SAMTA\Desktop\Julie\table 3 a,b,c12thApril twao way anova
 data file.sav
```

Between-Subjects Factors

		Value Label	N
HormoneType	1.00	IAA	20
	2.00	IBA	20
	3.00	NAA	20
Concentration	1.00	0.2	12
	2.00	0.4	12
	3.00	0.6	12
	4.00	0.8	12
	5.00	1.0	12

Descriptive Statistics

Dependent Variable: BasalCallus

HormoneType	Concentration	Mean	Std. Deviation	N
IAA	0.2	.0000	.00000	4
	0.4	.0000	.00000	4
	0.6	3.2175	.77937	4
	0.8	5.5700	.43344	4
	1.0	17.4325	2.10905	4
	Total	5.2440	6.67602	20
IBA	0.2	.0000	.00000	4
	0.4	.0000	.00000	4
	0.6	.0000	.00000	4
	0.8	.0000	.00000	4
	1.0	7.0575	1.46170	4
	Total	1.4115	2.95400	20
NAA	0.2	.0000	.00000	4
	0.4	7.1750	1.45177	4
	0.6	10.1800	1.66022	4
	0.8	12.0550	.69092	4
	1.0	21.8925	.35818	4
	Total	10.2605	7.36179	20
Total	0.2	.0000	.00000	12
	0.4	2.3917	3.61316	12
	0.6	4.4658	4.53978	12
	0.8	5.8750	5.16280	12
	1.0	15.4608	6.63063	12
	Total	5.6387	6.92582	60

Levene's Test of Equality of Error Variances[a]

Dependent Variable: BasalCallus

F	df1	df2	Sig.
4.425	14	45	.000

Tests the null hypothesis that the error variance of the dependent variable is equal across groups.

a. Design: Intercept + HormoneType + Concentration + HormoneType * Concentration

Tests of Between-Subjects Effects

Dependent Variable: BasalCallus

Source	Type III Sum of Squares	df	Mean Square	F	Sig.
Corrected Model	2791.507[a]	14	199.393	232.763	.000
Intercept	1907.674	1	1907.674	2226.932	.000
HormoneType	787.721	2	393.860	459.775	.000
Concentration	1682.927	4	420.732	491.143	.000
HormoneType * Concentration	320.859	8	40.107	46.820	.000
Error	38.549	45	.857		
Total	4737.729	60			
Corrected Total	2830.056	59			

a. R Squared = .986 (Adjusted R Squared = .982)

Estimated Marginal Means

1. Grand Mean

Dependent Variable: BasalCallus

Mean	Std. Error	95% Confidence Interval	
		Lower Bound	Upper Bound
5.639	.119	5.398	5.879

2. HormoneType

Estimates

Dependent Variable: BasalCallus

HormoneType	Mean	Std. Error	95% Confidence Interval	
			Lower Bound	Upper Bound
IAA	5.244	.207	4.827	5.661
IBA	1.412	.207	.995	1.828
NAA	10.261	.207	9.844	10.677

Pairwise Comparisons

Dependent Variable: BasalCallus

(I) HormoneType	(J) HormoneType	Mean Difference (I-J)	Std. Error	Sig.[b]	95% Confidence ...[b]
					Lower Bound
IAA	IBA	3.833*	.293	.000	3.243
	NAA	-5.017*	.293	.000	-5.606
IBA	IAA	-3.833*	.293	.000	-4.422
	NAA	-8.849*	.293	.000	-9.438
NAA	IAA	5.017*	.293	.000	4.427
	IBA	8.849*	.293	.000	8.260

Pairwise Comparisons

Dependent Variable: BasalCallus

(I) HormoneType	(J) HormoneType	95% Confidence ...[b]
		Upper Bound
IAA	IBA	4.422
	NAA	-4.427
IBA	IAA	-3.243
	NAA	-8.260
NAA	IAA	5.606
	IBA	9.438

Based on estimated marginal means

*. The mean difference is significant at the .05 level.

b. Adjustment for multiple comparisons: Least Significant Difference (equivalent to no adjustments).

Univariate Tests

Dependent Variable: BasalCallus

	Sum of Squares	df	Mean Square	F	Sig.
Contrast	787.721	2	393.860	459.775	.000
Error	38.549	45	.857		

The F tests the effect of HormoneType. This test is based on the linearly independent pairwise comparisons among the estimated marginal means.

3. Concentration

Estimates

Dependent Variable: BasalCallus

Concentration	Mean	Std. Error	95% Confidence Interval	
			Lower Bound	Upper Bound
0.2	-1.113E-013	.267	-.538	.538
0.4	2.392	.267	1.854	2.930
0.6	4.466	.267	3.928	5.004
0.8	5.875	.267	5.337	6.413
1.0	15.461	.267	14.923	15.999

Pairwise Comparisons

Dependent Variable: BasalCallus

(I) Concentration	(J) Concentration	Mean Difference (I-J)	Std. Error	Sig.[b]	95% Confidence ...[b]
					Lower Bound
0.2	0.4	-2.392*	.378	.000	-3.153
	0.6	-4.466*	.378	.000	-5.227
	0.8	-5.875*	.378	.000	-6.636
	1.0	-15.461*	.378	.000	-16.222
0.4	0.2	2.392*	.378	.000	1.631
	0.6	-2.074*	.378	.000	-2.835
	0.8	-3.483*	.378	.000	-4.244
	1.0	-13.069*	.378	.000	-13.830
0.6	0.2	4.466*	.378	.000	3.705
	0.4	2.074*	.378	.000	1.313
	0.8	-1.409*	.378	.001	-2.170
	1.0	-10.995*	.378	.000	-11.756
0.8	0.2	5.875*	.378	.000	5.114
	0.4	3.483*	.378	.000	2.722
	0.6	1.409*	.378	.001	.648
	1.0	-9.586*	.378	.000	-10.347

Pairwise Comparisons

Dependent Variable: BasalCallus

(I) Concentration	(J) Concentration	95% Confidence [b] ... Upper Bound
0.2	0.4	-1.631
	0.6	-3.705
	0.8	-5.114
	1.0	-14.700
0.4	0.2	3.153
	0.6	-1.313
	0.8	-2.722
	1.0	-12.308
0.6	0.2	5.227
	0.4	2.835
	0.8	-.648
	1.0	-10.234
0.8	0.2	6.636
	0.4	4.244
	0.6	2.170
	1.0	-8.825

Pairwise Comparisons

Dependent Variable: BasalCallus

(I) Concentration	(J) Concentration	Mean Difference (I-J)	Std. Error	Sig.[b]	95% Confidence [b] ... Lower Bound
1.0	0.2	15.461*	.378	.000	14.700
	0.4	13.069*	.378	.000	12.308
	0.6	10.995*	.378	.000	10.234
	0.8	9.586*	.378	.000	8.825

Pairwise Comparisons

Dependent Variable: BasalCallus

(I) Concentration	(J) Concentration	95% Confidence [b] ... Upper Bound
1.0	0.2	16.222
	0.4	13.830
	0.6	11.756
	0.8	10.347

Based on estimated marginal means

*. The mean difference is significant at the .05 level.

b. Adjustment for multiple comparisons: Least Significant Difference (equivalent to no adjustments).

Univariate Tests

Dependent Variable: BasalCallus

	Sum of Squares	df	Mean Square	F	Sig.
Contrast	1682.927	4	420.732	491.143	.000
Error	38.549	45	.857		

The F tests the effect of Concentration. This test is based on the linearly independent pairwise comparisons among the estimated marginal means.

4. HormoneType * Concentration

Dependent Variable: BasalCallus

				95% Confidence Interval	
HormoneType	Concentration	Mean	Std. Error	Lower Bound	Upper Bound
IAA	0.2	-1.071E-013	.463	-.932	.932
	0.4	-1.036E-013	.463	-.932	.932
	0.6	3.218	.463	2.285	4.150
	0.8	5.570	.463	4.638	6.502
	1.0	17.433	.463	16.500	18.365
IBA	0.2	-1.071E-013	.463	-.932	.932
	0.4	-1.027E-013	.463	-.932	.932
	0.6	-1.036E-013	.463	-.932	.932
	0.8	.000	.463	-.932	.932
	1.0	7.058	.463	6.125	7.990
NAA	0.2	-1.213E-013	.463	-.932	.932
	0.4	7.175	.463	6.243	8.107
	0.6	10.180	.463	9.248	11.112
	0.8	12.055	.463	11.123	12.987
	1.0	21.893	.463	20.960	22.825

Post Hoc Tests

HormoneType

Multiple Comparisons

Dependent Variable: BasalCallus

Tukey HSD

(I) HormoneType	(J) HormoneType	Mean Difference (I-J)	Std. Error	Sig.	95% ... Lower Bound
IAA	IBA	3.8325*	.29268	.000	3.1231
	NAA	-5.0165*	.29268	.000	-5.7259
IBA	IAA	-3.8325*	.29268	.000	-4.5419
	NAA	-8.8490*	.29268	.000	-9.5584
NAA	IAA	5.0165*	.29268	.000	4.3071
	IBA	8.8490*	.29268	.000	8.1396

Multiple Comparisons

Dependent Variable: BasalCallus

Tukey HSD

(I) HormoneType	(J) HormoneType	95% ... Upper Bound
IAA	IBA	4.5419
	NAA	-4.3071
IBA	IAA	-3.1231
	NAA	-8.1396
NAA	IAA	5.7259
	IBA	9.5584

Based on observed means.
The error term is Mean Square(Error) = .857.

*. The mean difference is significant at the .05 level.

Homogeneous Subsets

BasalCallus

Tukey HSD[a,b]

HormoneType	N	Subset 1	Subset 2	Subset 3
IBA	20	1.4115		
IAA	20		5.2440	
NAA	20			10.2605
Sig.		1.000	1.000	1.000

Means for groups in homogeneous subsets are displayed.
Based on observed means.
The error term is Mean Square(Error) = .857.

a. Uses Harmonic Mean Sample Size = 20.000.

b. Alpha = .05.

Concentration

Multiple Comparisons

Dependent Variable: BasalCallus

Tukey HSD

(I) Concentration	(J) Concentration	Mean Difference (I-J)	Std. Error	Sig.	95% ... Lower Bound
0.2	0.4	-2.3917*	.37785	.000	-3.4653
	0.6	-4.4658*	.37785	.000	-5.5395
	0.8	-5.8750*	.37785	.000	-6.9487
	1.0	-15.4608*	.37785	.000	-16.5345
0.4	0.2	2.3917*	.37785	.000	1.3180
	0.6	-2.0742*	.37785	.000	-3.1478
	0.8	-3.4833*	.37785	.000	-4.5570
	1.0	-13.0692*	.37785	.000	-14.1428
0.6	0.2	4.4658*	.37785	.000	3.3922
	0.4	2.0742*	.37785	.000	1.0005
	0.8	-1.4092*	.37785	.005	-2.4828
	1.0	-10.9950*	.37785	.000	-12.0687
0.8	0.2	5.8750*	.37785	.000	4.8013
	0.4	3.4833*	.37785	.000	2.4097
	0.6	1.4092*	.37785	.005	.3355
	1.0	-9.5858*	.37785	.000	-10.6595
1.0	0.2	15.4608*	.37785	.000	14.3872
	0.4	13.0692*	.37785	.000	11.9955
	0.6	10.9950*	.37785	.000	9.9213
	0.8	9.5858*	.37785	.000	8.5122

Multiple Comparisons

Dependent Variable: BasalCallus

Tukey HSD

(I) Concentration	(J) Concentration	95% ... Upper Bound
0.2	0.4	-1.3180
	0.6	-3.3922
	0.8	-4.8013
	1.0	-14.3872
0.4	0.2	3.4653
	0.6	-1.0005
	0.8	-2.4097
	1.0	-11.9955
0.6	0.2	5.5395
	0.4	3.1478
	0.8	-.3355
	1.0	-9.9213
0.8	0.2	6.9487
	0.4	4.5570
	0.6	2.4828
	1.0	-8.5122
1.0	0.2	16.5345
	0.4	14.1428
	0.6	12.0687
	0.8	10.6595

Based on observed means.
The error term is Mean Square(Error) = .857.

*. The mean difference is significant at the .05 level.

Homogeneous Subsets

BasalCallus

Tukey HSD[a,b]

Concentration	N	Subset				
		1	2	3	4	5
0.2	12	.0000				
0.4	12		2.3917			
0.6	12			4.4658		
0.8	12				5.8750	
1.0	12					15.4608
Sig.		1.000	1.000	1.000	1.000	1.000

Means for groups in homogeneous subsets are displayed.
Based on observed means.
The error term is Mean Square(Error) = .857.

a. Uses Harmonic Mean Sample Size = 12.000.

b. Alpha = .05.
